# Somatostatin Interneurons of the Insula Mediate QR2-Dependent Novel Taste Memory Enhancement

**DOI:** 10.1523/ENEURO.0152-21.2021

**Published:** 2021-09-28

**Authors:** Nathaniel L. Gould, Sailendrakumar Kolatt Chandran, Haneen Kayyal, Efrat Edry, Kobi Rosenblum

**Affiliations:** 1 Sagol Department of Neuroscience, University of Haifa, Haifa 3498838, Israel; 2 Center for Gene Manipulation in the Brain, University of Haifa, Haifa 3498838, Israel

**Keywords:** consolidation, insula, memory, novel, QR2, taste

## Abstract

Forming long-term memories is crucial for adaptive behavior and survival in changing environments. The molecular consolidation processes which underlie the formation of these long-term memories are dependent on protein synthesis in excitatory and SST-expressing neurons. A centrally important, parallel process to this involves the removal of the memory constraint quinone reductase 2 (QR2), which has been recently shown to enhance memory consolidation for novel experiences in the cortex and hippocampus, via redox modulation. However, it is unknown within which cell type in the cortex removal of QR2 occurs, nor how this affects neuronal function. Here, we use novel taste learning in the mouse anterior insular cortex (aIC) to show that similarly to mRNA translation, QR2 removal occurs in excitatory and SST-expressing neurons. Interestingly, both novel taste and QR2 inhibition reduce excitability specifically within SST, but not excitatory neurons. Furthermore, reducing QR2 expression in SST, but not in PV or excitatory neurons, is sufficient to enhance taste memory. Thus, QR2 mediated intrinsic property changes of SST interneurons in the aIC is a central removable factor to allow novel taste memory formation. This previously unknown involvement of QR2 and SST interneurons in resetting aIC activity hours following learning, describes a molecular mechanism to define cell circuits for novel information. Therefore, the QR2 pathway in SST interneurons provides a fresh new avenue by which to tackle age-related cognitive deficits, while shedding new light onto the functional machinations of long-term memory formation for novel information.

## Significance Statement

Quinone reductase 2 (QR2) removal from the cortex and hippocampus demarcates novelty, and facilitates memory formation for the associated events to a novel experience. The newly described QR2 pathway, and the circuit that is responsible for late consolidation of novel internal representations, are both currently being investigated and are not yet wholly understood. Presently, a hitherto unknown phenomenon of reduced SST activity in the hours following novel taste learning via QR2 removal is shown, providing a molecular mechanism to define a brain state enacted by distinct neuronal populations. This provides both new molecular and cellular information regarding an important time period in memory formation, and also describes the key cell types involved in the underlying circuit, which is as yet unexplored.

## Introduction

Learning new information about the environment is critical for behavioral adaptation and survival (Squire et al., 2015a; Eichenbaum, 2017). Novel taste learning, necessary to identify safe food, is predicated on the release of acetylcholine (ACh) in the anterior insular cortex (aIC), the brain region which subserves taste learning and memory (Gutiérrez et al., 2003; Yiannakas and Rosenblum, 2017). For decades, researchers in the field of molecular mechanisms underlying memory consolidation and synaptic plasticity focused on protein synthesis regulation (Davis and Squire, 1984; Santini et al., 2014). Recently, we described a central pathway downstream of ACh in the aIC that is necessary for novel taste memory formation, in which quinone reductase 2 (QR2) acts as a removable constraint, in parallel and possibly separately from proteostasis (Rappaport et al., 2015; Gould et al., 2020b). Although reduced QR2 expression may affect the cell in a number of ways (Olshina et al., 2020; Sonavane et al., 2020), we identified that by removing QR2, which continuously generates physiological levels of reactive oxygen species (ROS), the aIC becomes less oxidized. Thus, levels of oxidized and deactivated voltage gated potassium channel Kv2.1 are reduced, hours following learning, presumably along with other redox sensitive cellular components. Ablation of this redox modulation pathway and novel taste memory formation, by scopolamine, is rescued by directly inhibiting the QR2 enzyme (Gould et al., 2020b). QR2 is therefore part of a necessary oxidative eustress mechanism that denotes a novel memory, distinguishing the internal representation formed for the experience compared with that of a familiar experience. However, the detailed cell type/s involved in this molecular process within the aIC are unknown.

The aIC is a complex cortical structure that associates external events with their visceral consequences and acts across several forms of learning and behavior (Gogolla, 2017). It is comprised of the granular aIC (gIC), disgranular aIC (dgIC), and agranular aIC (agIC), in a dorsoventral arrangement, within which reside a wide variety of cell types. Part of the current effort in understanding memory formation lies in placing molecular classification to cell types within subregions that are themselves functionally classified (Sharma et al., 2020; Shrestha et al., 2020). One would assume that different neurons act in discrete, particular fashion, to accommodate cellular and molecular processes that presumably underlie the formation, maintenance or retrieval of an internal representation (Kepecs and Fishell, 2014). The QR2 pathway has been shown to act 3 h following novel taste learning and, therefore, presents a target to define cells involved in the late phase of memory consolidation (Alberini and Kandel, 2014). Although the downstream molecular targets of QR2 are still poorly understood, we found that in the hippocampus, QR2 removal 3 h following novelty exerts an inhibitory effect within inhibitory neurons, by hyperpolarizing their resting membrane potential (RMP; Gould et al., 2020a). This is sufficient to induce a hippocampal “novelty state,” allowing the enhanced memory encoding that is associated with contextual novelty. By locating QR2 removal in the aIC, we may start to discover the effect of this newly described molecular pathway on the cell and circuit level which subserve novel taste memory formation, which currently represents a major open question. Here, we found that the QR2 pathway is primarily involved in aIC inhibitory neurons, and in particular that it is activated within SST interneurons across all subregions of the aIC. Additionally, we found that in correlation to QR2 pathway activation 3 h following novel taste consumption, SST interneurons show reduced intrinsic excitability and an increase in medium after-hyperpolarization (mAHP), an effect strongly replicated by QR2 inhibition specifically in these cells. Furthermore, reduced QR2 expression in SST interneurons alone is sufficient to enhance taste memory consolidation. This therefore connects the QR2 pathway to SST interneuron function, indicating the importance of both these molecular and cellular components within the emerging circuitry of normal novel taste memory formation.

## Materials and Methods

### Subjects

Male and female mice, 8–16 weeks old, weighing 20–30 g from the following strains were used: C57BL/6 (Envigo), Gad2-IRES-Cre, SST-IRES-Cre, B6 PV-Cre and VIP-IRES-Cre (The Jackson Laboratory stock #010802, #013044, #008069, and #010908, respectively). The mice were kept in a temperature-controlled facility (22–24 °C), on a 12/12 h light/dark cycle (light phase 7 A.M. to 7 P.M.) at the University of Haifa, with water and food provided *ad libitum*. Experimentation was approved by the University of Haifa Animal Care and Use Committee (license numbers: 437/16, 487/17, 488/17, and 631/19). Before experimentation, mice were allowed 7 d of acclimatization. Animals were handled in accordance with University of Haifa practices and standards, in compliance with the National Institutes of Health guidelines for the ethical treatment of animals.

### Animal behavior

Mice were taught to drink water from pipettes (2 ml/pipette) once a day for 20 min, over 3 d. They were then given pipettes containing a novel taste (0.5% saccharin). Following novel taste consumption, animals were killed 3 h later, at the time point when QR2 mRNA expression reduction is best measured (Rappaport et al., 2015). To assess long-term memory of incidental novel taste learning, as indicated by novel taste preference using a choice test, mice were given a pipette of water and a pipette of the novel taste (2 ml each), 48 h after the novel taste was first consumed. Memory of the novel taste was determined by calculating a preference index, by dividing the volume of novel taste by the total volume consumed, [novel taste/(novel taste + water)] × 100 (Rosenblum et al., 1993).

### AAV plasmids and production of recombinant AAV vectors

QR2 shRNA sequences (shNQO2 and scrambled control) were cloned into pAAV-Sico-Red plasmid, which was a gift from Eun Mi Hwang (Addgene plasmid #84882; http://n2t.net/addgene:84882; RRID:Addgene_84882), using conventional cloning techniques. HEK293FT cells were seeded at 25–35% confluence to produce AAV vectors, and were transfected 24 h later. The plasmids encoding AAV rep, cap of AAV1 and AAV2, and a vector plasmid for the rAAV cassette, expressing the relevant shRNA, were applied using the PEI method (Grimm et al., 2003; Edry et al., 2011). 72 h after transfection, the cells and medium were harvested, pelleted by centrifugation (300 × *g*), resuspended in lysis solution (150 mm NaCl and 50 mm Tris-HCl; pH 8.5 with NaOH) and lysed via X3 freeze-thaw cycles. Treatment of the crude lysate with 250 U benzonase (Sigma) per 1 ml of lysate at 37°C for 1.5 h was then done, to degrade genomic and unpackaged AAV DNA, followed by centrifugation at 3000 × *g* for 15 min to pellet cell debris. Heparin-agarose columns were used to purify the virus particles from the supernatant, which were then washed with PBS and concentrated with Amicon (Merck Millipore) columns. The resultant viral suspension was then aliquoted and stored at −80°C. Viral titer was determined by qPCR. AAV vectors used for injections had genomic titers ranging between 2 × 10^10^ and 5 ×10^10^ genome copies per milliliter (gc/ml). The AAV p315 (ssAAV-1/2-mCaMKIIα-EGFP_2A_iCre-WPRE-SV40p(A), physical titer 4.6 × 10^12^ vg/ml) was purchased from the University of Zurich Viral Vector Facility (https://vvf.ethz.ch/), to express Cre under the CamKII promoter.

### Surgeries and viral vector injection

Mice were anesthetized with ketamine and domitor (0.5 mg/kg each), given an analgesic (norocarp, 0.5 mg/kg) and 40 min later were placed in a robot stereotaxic device (Neurostar). The scalp was opened, and bregma and λ were marked as reference points for the drilling and injection site (coordinates for aIC relative to bregma: AP 0.86 mm, ML ±3.4 mm, DV 4 mm). Following syringe (Hamilton) insertion, 5 min were given before 0.2- to 0.8-μl virus was injected, at 0.05 μl/min using StereoDrive and InjectoMate software (Neurostar). The syringe was then left in the injection site for a further 10 min to prevent capillary motion of the virus from the site. Upon completion, incisions were dressed with 5% synthomycine ointment (Rekah), sealed shut with Vetbond (3M), 0.5 mg/kg enrofloxacin (Baytril) was given and mice were administered 0.25 mg/kg atipamezole hydrochloride (Antisedan) and placed in a heated recovery cage for 1–2 h. A month-long period of mouse recovery and virus expression was then given, following which experimentation began.

### Brain dissection and tissue preparation

Mice were swiftly killed by cervical dislocation, brains were removed and instantly flash frozen with liquid nitrogen and stored in −80°C. Brain sections and tissue punches were conducted in a Leica CM 1950 cryostat. Samples for qPCR were dissected from 1-mm-thick coronal slices (from bregma: AP 1.18 mm, ML ±3 mm, DV 3.6 mm, to bregma: AP 0.14 mm, ML ±3.6 mm, DV 4.1 mm, using Franklin and Paxinos, 2007) with a 1-mm diameter tissue punching device. Samples for RNAscope were sliced in 20-μm sections, from bregma AP 1.25–0.25 mm, mounted directly onto SuperFrost Ultra Plus Adhesion slides (Thermo Fisher Scientific) and kept in −80°C.

### RNA extraction and qPCR

RNA extraction, cDNA generation and qPCR were conducted as previously described (Gould et al., 2020b). Briefly, RNA was extracted using Tri reagent. cDNA was then synthesized using the Applied Biosystems (Thermo Fisher Scientific) High Capacity cDNA Reverse Transcription (RT) kit, according to manufacturer’s instructions. Taqman (Thermo Fisher Scientific) primers were then used to detect QR2 (Mm01332867_m1), GAPDH (Mm99999915_g1) and HPRT (Mm00446968_m1). A custom primer was made for the measurement of mCherry (forward primer, GGCGCCTACAACGTCAACAT; reverse primer, TCGGCGCGTTCGTACTGT; probe, ACAACGAGGACTACACCAT). Relative quantitation was done using the 2^ΔΔCt^ method (Livak and Schmittgen, 2001).

### RNAscope and image processing

Over two separate experiments, mice received either a familiar (water, or seventh exposure to saccharin) or a novel taste (first exposure to saccharin), were killed 3 h later and fresh frozen brains were sliced into 20-μm sections in a Leica CM 1950 cryostat, and then mounted onto SuperFrost Ultra Plus Adhesion slides (Thermo Fisher Scientific). RNAscope (ACD) fluorescent *in situ* hybridization (FISH) was then conducted as previously described (Golden et al., 2019; Gould et al., 2020a) by an experimenter blind to the identity of the groups, according to the manufacturer’s instructions, using probes for Gad1, Slc17a7 (vGlut), Sst, Pvalb (PV), and Vip to mark out cells and Nqo2 (QR2) to measure changes to QR2 within these cells. Upon completion, slices were mounted using ProLong Gold antifade (Thermo Fisher Scientific). Slides were then left overnight in the dark, at room temperature, and were moved to 4°C refrigeration for continued storage. Images of the fluorescently labeled cells and QR2 were acquired with an Olympus IX81 microscope and Cell Sense software. Images were taken such as to form a z stack of three layers, 1.5 μm apart. The stacks were then used to deconvolute the images to remove background, nonspecific signal. The deconvoluted images were then exported as BigTIFF and further processing was conducted using FIJI (https://imagej.net/Fiji), where regions of interest (ROIs) were manually drawn to denote aIC subregions, using the Allen Mouse Brain Atlas (http://mouse.brain-map.org/) for reference (Dong, 2008). Standardized background noise reduction was then done using the BioVoxxel ‘Convoluted Background Subtraction’ FIJI plugin, with Gaussian filter selected and set at a radius of 100 μm, and images were then converted into binary masks (0–255). The FIJI binary process Erode was then used to remove remaining signal background noise, speckles and debris, until almost only whole bodied cells were left. The remaining cell bodies were then brought back to their original size using the Dilate FIJI binary function. When co probing for GAD and vGlut, GAD masks were subtracted from vGlut to prevent double counting. Masked, binary representations of the cells (GAD, vGlut, PV, SST, and VIP) were then selected, and ROIs were generated for each cell mask, using the FIJI Analyze Particles function and providing a minimum size (100–200 μm^2^) to avoid counting any remaining debris or incomplete cell segments and other artifacts. The fluorescent QR2 signal was then measured within these cell-defined ROIs, using the FIJI Command Manager Measure function, and data were collated into relevant bins (e.g., aIC subregion, layer). QR2 signal within these cells was then averaged for each animal, and each animals mean QR2 expression per brain area and cell type was then used. In order to minimize signal variation because of technical factors existing between experiments (e.g., probe shelf life, microscope light intensity), the average QR2 expression from every animal in each of the groups (i.e., novel, familiar) was divided by the control (familiar water) group mean QR2 signal, in each experiment separately. This normalized QR2 expression from each separate experiment was then combined, and a comparison was done between experimental groups (i.e., novel or familiar). When comparing relative contribution of QR2 signal from within excitatory or inhibitory cell types, cells were pooled from all animals in either familiar or novel taste groups and binned into either vGlut or GAD positive pools. The sum total QR2 signal from each of the pooled cell types was then divided by the total cell number within each experimental group (familiar or novel taste). Then, the reduction in QR2 signal was calculated by deducting the novel taste group QR2 signal, from each cell type, from that of the familiar taste group.

### Electrophysiology

#### Tissue preparation

Mice were killed 3 h following water or saccharin consumption by decapitation, following anesthesia with isoflurane. Brain slices were cut at 300 μm in coronal sections using a vibratome (Campden-1000) in ice chilled cutting solution (110 mm sucrose, 60 mm NaCl, 3 mm KCl, 1.25 mm NaH_2_PO_4_, 28 mm NaHCO_3_, 0.5 mm CaCl_2_, 7 mm MgCl_2_, 5 mm D-glucose, and 0.6 mm ascorbate, Sigma-Aldrich). The slices were placed in artificial CSF (ACSF; 125 mm NaCl, 2.5 mm KCl, 1.25 mm NaH_2_PO_4_, 25 mm NaHCO_3_, 25 mm D-glucose, 2 mm CaCl_2_, and 1 mm MgCl_2_, Sigma-Aldrich) for a 30 min recovery period at 37°C and then kept at room temperature for at least an additional 30 min before electrophysiological recording. Throughout, ACSF was continually gassed, using carbogen (O_2_ 95%, CO_2_ 5%).

#### Intracellular whole-cell recording

Intrinsic properties were measured as previously reported (Chakraborty et al., 2017). Slices were placed in a recording chamber, following a 1-h recovery period in ACSF, and kept at 32–34°C while continuously being washed in carbogenated ACSF (2 ml/min). Pyramidal cells were identified using differential interference contrast microscopy (Olympus BX51-WI), with 10× or 40× water immersion objectives. Images were acquired with a monitor using a charge-coupled device (CCD) camera (Dage MTI). In SST interneurons expressing Cre, a Cre-dependent mCherry reporter was used to positively identify the fluorescently labeled SST interneurons. A Multiclamp Axopatch 200B amplifier was used for recordings, which were digitized using a Digidata 1440 (Molecular Devices). Borosilicate glass pipettes (3–5 M) were used with a P-1000 electrode puller (Sutter Instruments) to make recording electrodes, which were then filled with 290 mOsm, pH 7.3, internal solution (130 mm K-gluconate, 5 mm KCl, 10 mm HEPES, 2.5 mm MgCl_2_, 0.6 mm EGTA, 4 mm Mg-ATP, 0.4 mm Na_3_GTP, and 10 mm phosphocreatine, Sigma-Aldrich). Recordings were done from soma of pyramidal neurons and SST interneurons expressing mCherry, from the aIC of C57BL/6 and SST Cre mice with 0.5 μm S29434 (synthesized at the G-INCPM, Weizmann Institute) in 0.5% DMSO, or vehicle, in the patch pipette. No online correction was done for liquid junction potentials (10 mV), and current clamp recordings were low-pass filtered at 10 kHz and sampled at 50 kHz. Only cells with resistance smaller than 20 MΩ were included, once compensation for pipette capacitance and series resistance was done.

#### Recording parameters

RMP was measured 10 s following commencement of whole-cell recording, done by rupturing the membrane directly under the recording pipette (<–50 mV). Injection of 500-ms, 50-pA current steps, from 50 to 400 pA, was used to measure neuron firing rate (while in current clamp, at the RMP of the cell). A hyperpolarizing current pulse (−150 pA) elicited a voltage response which was used to calculate the input resistance (Rin). A sag ratio was calculated using [(1 – ΔV_SS_/ΔV _max_) × 100%] of the voltage response to –150 pA, as previously described (Song et al., 2015). Membrane time constant was determined by a single exponential fit to the first 100 ms of the raising phase of the neurons response to a 1-s, –150-pA hyperpolarization step.

For 10 ms, a series of brief, depolarizing currents were injected in 10-pA increments to measure single action potentials, following an initial assessment of the current required to elicit an action potential following 15 ms of current injection, using 50-pA steps. At 5 ms, the first action potential was analyzed. A dV/dt curve was made for the action potential, using the 30-V/s point in the rising slope as threshold (Chakraborty et al., 2017). The duration of the action potential was measured at the half-amplitude point of the spike. The equipotential point of the threshold and the spike peak were used to measure the spike amplitude. mAHP was measured with 3-nA high amplitude somatic current injections 3 s long, to initiate time locked action potential trains at 50 Hz (10–50 Hz, 1 or 3 s). A prolonged (20 s) AHP was caused by the action potential trains, the amplitudes and integrals of which increased in proportion to the number of spikes in the elicited train. AHP was then measured using the equipotential point of the threshold and the anti-peak of the same spike (Gulledge et al., 2013). During the duration of the experiment, series resistance, membrane capacitance and Rin were monitored, with exclusion of data showing ≥30% changes in these parameters.

### Statistical analysis

Male and female subjects were randomly allocated to experimental groups. Group size range estimation was based on previously published results using similar methods, as well as an online power calculator (https://www.stat.ubc.ca/∼rollin/stats/ssize/n2.html). Data obtained were tested for normality, using Shapiro–Wilk normality test. Normally distributed data were then analyzed by unpaired Student’s *t* test, one-way ANOVA or repeated measures two-way ANOVA, followed by Tukey’s or Sidak’s *post hoc* analysis. For non-parametric analysis, Mann–Whitney tests or Kruskal–Wallis followed by Dunn’s multiple comparisons tests were conducted. All data are presented as means with SEM. All descriptive statistics, normality tests, parametric and non-parametric tests were conducted using GraphPad Prism 7 software.

## Results

### QR2 expression is reduced in inhibitory neurons of the aIC 3 h following novel taste learning

**Figure 1.**
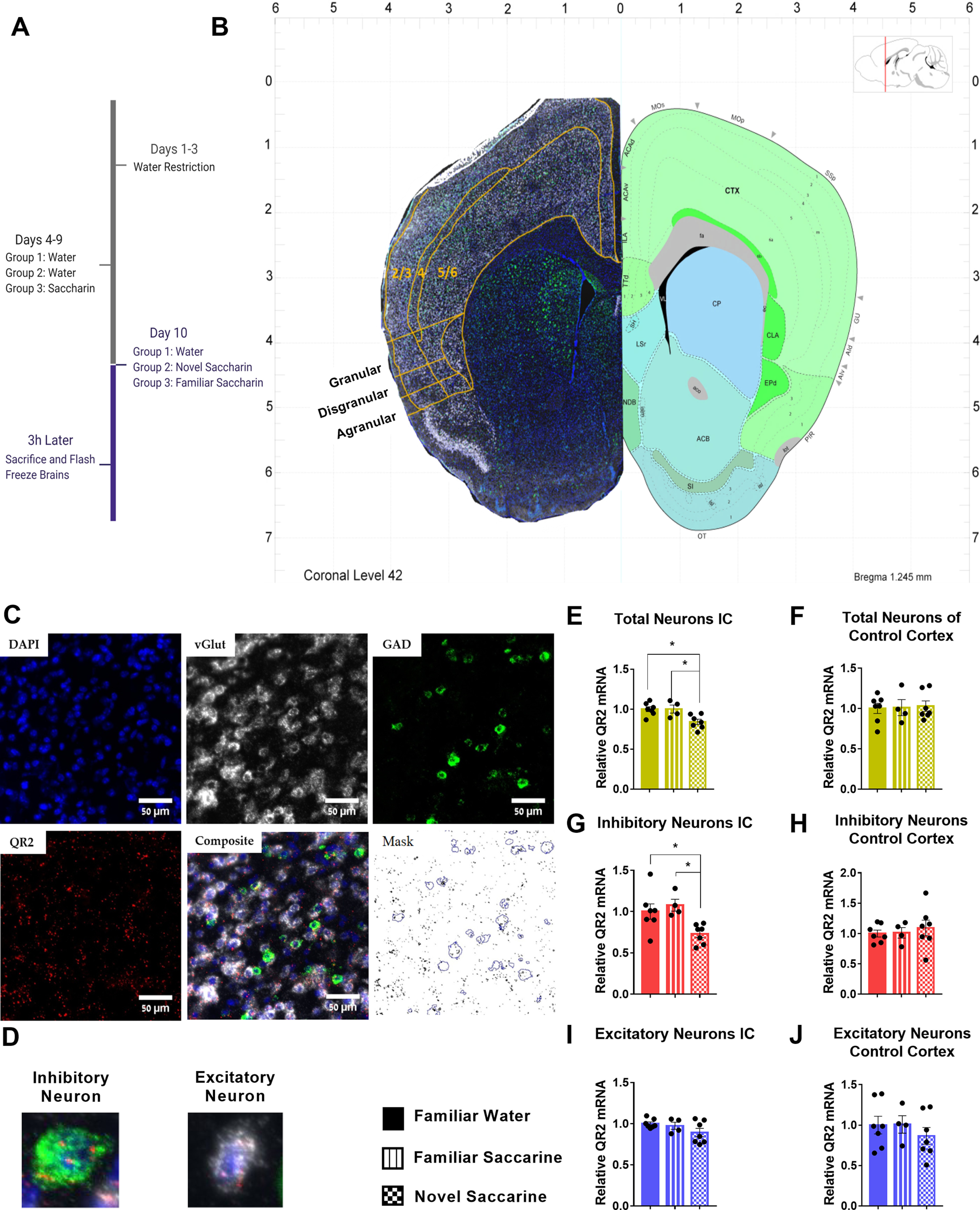
QR2 expression is reduced in inhibitory neurons of the aIC 3 h following novel taste learning. ***A***, Mice were allocated randomly to three groups which consisted of water, familiarized saccharin and novel saccharin, and were killed 3 h following consumption. ***B***, Brains of mice killed 3 h following familiar or novel taste consumption were hybridized using RNAscope FISH probes. Relative QR2 mRNA expression in neurons was then measured and compared with familiar taste (water) controls. Brain atlas image credit: Allen Institute. ***C***, Neurons were marked for vGlut (excitatory cells) or GAD (inhibitory cells) and QR2 mRNA was measured within cell outlines denoted by the cell markers. ***D***, Representative images of an inhibitory (green) and an excitatory (white) neuron, expressing QR2 (red). ***E***, QR2 mRNA FISH-labeled expression levels are reduced in the aIC following novel taste compared with either familiar tastes (familiar water 1 ± 0.030 AU, *n* = 7; familiar saccharin 1.001 ± 0.050 AU, *n* = 4; novel saccharin 0.841 ± 0.032 AU, *n* = 7; one-way ANOVA, *F*_(2,15)_ = 7.107, *p* = 0.0067; Tukey’s multiple comparisons *post hoc* test, familiar water vs familiar saccharin, *p* > 0.9999; familiar water vs novel saccharin, *p* = 0.0119; familiar saccharin vs novel saccharin, *p* = 0.0310). ***F***, No change to QR2 mRNA FISH-labeled expression levels were detected in the somatosensory cortex between the groups (familiar water 1.002 ± 0.063 AU, *n* = 7; familiar saccharin 1.011 ± 0.099 AU, *n* = 4; novel saccharin 1.028 ± 0.064 AU, *n* = 7; one-way ANOVA, *F*_(2,15)_ = 0.04,115, *p* = 0.9598). ***G***, A reduction in QR2 mRNA FISH-labeled expression levels is seen in inhibitory neurons (familiar water 1 ± 0.092 AU, *n* = 7; familiar saccharin 1.077 ± 0.071 AU, *n* = 4; novel saccharin 0.730 ± 0.044 AU, *n* = 7; one-way ANOVA, *F*_(2,15)_ = 5.856, *p* = 0.0132; Tukey’s multiple comparisons *post hoc* test, familiar water vs familiar saccharin, *p* = 0.7852; familiar water vs novel saccharin, *p* = 0.0373; familiar saccharin vs novel saccharin, *p* = 0.0224). ***H***, No change to QR2 mRNA FISH-labeled expression levels were detected in inhibitory neurons of the somatosensory cortex between the groups (familiar water 1 ± 0.057 AU, *n* = 7; familiar saccharin 1.011 ± 0.088 AU, *n* = 4; novel saccharin 1.091 ± 0.128 AU, *n* = 7; one-way ANOVA, *F*_(2,15)_ = 0.2639, *p* = 0.7716). ***I***, QR2 mRNA FISH-labeled expression levels are not significantly reduced in excitatory neurons (familiar water 1 ± 0.020 AU, *n* = 7 mice; familiar saccharin 0.973 ± 0.044 AU, *n* = 4; novel saccharin 0.891 ± 0.050 AU, *n* = 7; one-way ANOVA, *F*_(2,15)_ = 2.187, *p* = 0.1467). ***J***, No change to QR2 mRNA FISH-labeled expression levels were detected in excitatory neurons of the somatosensory cortex between the groups (familiar water 1 ± 0.109 AU, *n* = 7; familiar saccharin 1.008 ± 0.108 AU, *n* = 4; novel saccharin 0.866 ± 0.103 AU, *n* = 7; one-way ANOVA, *F*_(2,15)_ = 0.5481, *p* = 0.5892). Data are shown as mean ± SEM; *p < 0.05.

In order to determine the cell type and location within the aIC from which QR2 is removed following novel taste consumption, mice received either a familiarized taste (seventh exposure to 0.5% saccharin), water (highly familiar), or a novel taste (first exposure to 0.5% saccharin; Fig. 1*A*). The mice were then killed 3 h later, the time point at which QR2 removal is most notable when measuring mRNA using qPCR (Rappaport et al., 2015; Gould et al., 2020b), and RNAscope FISH was used on freshly frozen brain slices (Venniro et al., 2017). QR2 mRNA levels were probed by a second, blinded experimenter within excitatory (vGlut) and inhibitory (GAD1) neurons in the aIC of all three groups of mice (Fig. 1*B–D*). Relative QR2 expression was derived by dividing the mean fluorescent signal of each mouse to that of the average QR2 signal measured in the control (familiar water) group. A significant reduction in QR2 mRNA expression was seen in the aIC following novel taste only (Fig. 1*E*). In the control, somatosensory cortex, no change was detected (Fig. 1*F*). QR2 expression reduction in the aIC was most distinctly detected in inhibitory neurons (Fig. 1*G*), whereas excitatory neurons displayed only insignificantly reduced expression (Fig. 1*I*), while no significant changes were seen in either cell type in the control cortex (Fig. 1*H*,*J*). These results therefore point to inhibitory neurons as the main cell type in which QR2 expression reduction can be seen in the whole aIC following novel taste.

### Reduced QR2 expression observed across the aIC is significant within inhibitory neurons in the granular subregion and in layers 2/3 within both excitatory and inhibitory neurons following novel taste learning

**Figure 2.**
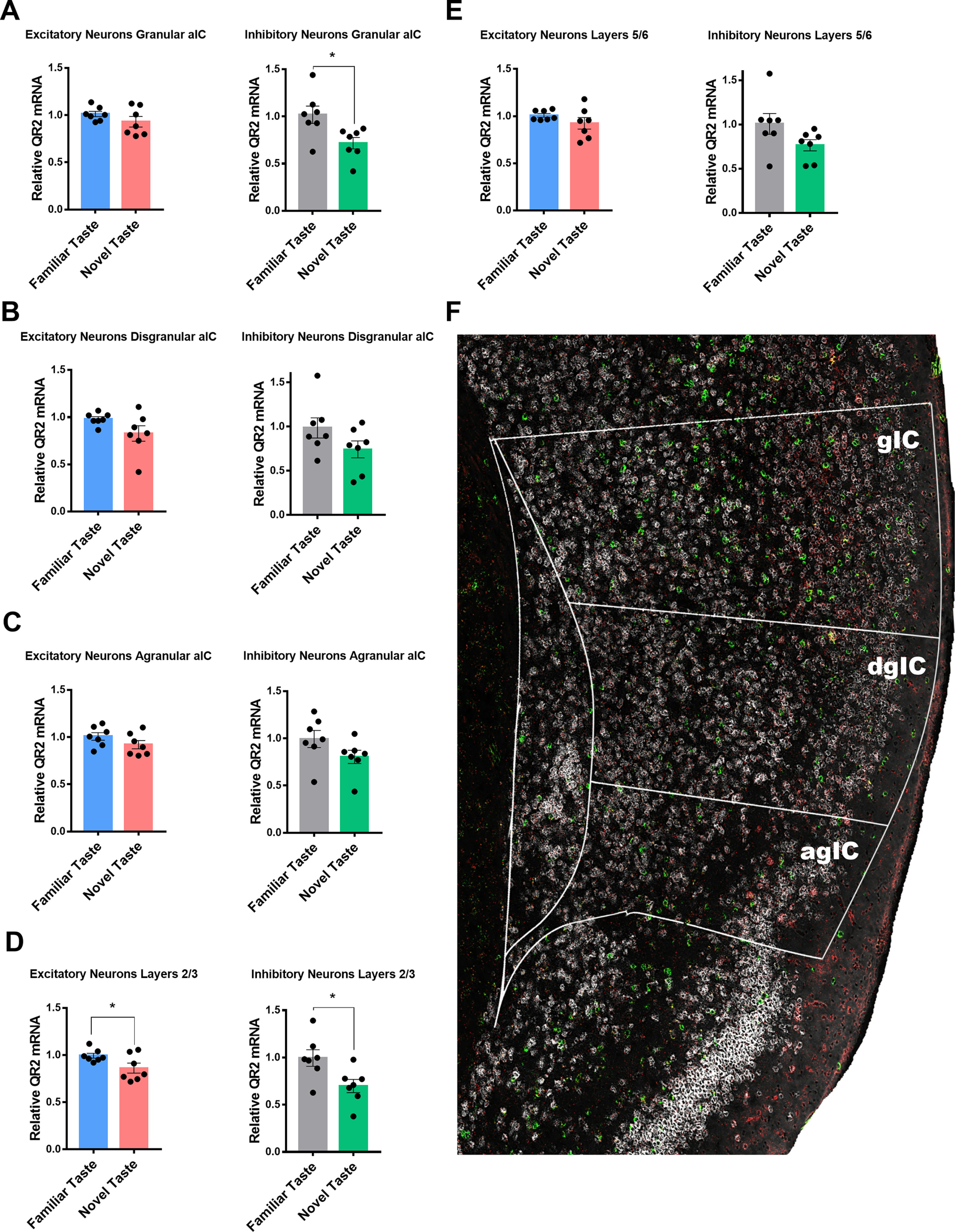
Reduced QR2 expression across the aIC is significant within inhibitory neurons in the granular subregion and in layers 2/3 within both excitatory and inhibitory neurons following novel taste learning. ***A***, QR2 mRNA FISH-labeled expression levels are not significantly reduced in the gIC in excitatory neurons (familiar taste 1.011 ± 0.028 AU, *n* = 7; novel taste 0.929 ± 0.055 AU, *n* = 7; Student’s *t* test, *t* = 1.304 df = 12, *p* = 0.2167) but show significant reduction in inhibitory neurons (familiar taste 1.018 ± 0.092 AU, *n* = 7; novel taste 0.716 ± 0.061 AU, *n* = 7; Student’s *t* test, *t* = 2.72 df = 12, *p* = 0.0186). ***B***, QR2 mRNA FISH-labeled expression levels are not significantly reduced in the dgIC in both excitatory (familiar taste 0.980 ± 0.024 AU, *n* = 7; novel taste 0.827 ± 0.081 AU, *n* = 7; Student’s *t* test, *t* = 1.801 df = 12, *p* = 0.0969) and inhibitory neurons (familiar taste 0.984 ± 0.114 AU, *n* = 7; novel taste 0.741 ± 0.095 AU, *n* = 7; Student’s *t* test, *t* = 1.636 df = 12, *p* = 0.1279). ***C***, QR2 mRNA FISH-labeled expression levels are not significantly reduced in the agIC in both excitatory (familiar taste 1.008 ± 0.039 AU, *n* = 7; novel taste 0.920 ± 0.043 AU, *n* = 7; Student’s *t* test, *t* = 1.478 df = 12, *p* = 0.1652) and inhibitory neurons (familiar taste 0.993 ± 0.090 AU, *n* = 7; novel taste 0.803 ± 0.069 AU, *n* = 7; Student’s *t* test, *t* = 1.658 df = 12, *p* = 0.1233). ***D***, QR2 mRNA FISH-labeled expression levels are reduced in layers 2/3 in the aIC in both excitatory (familiar taste 0.993 ± 0.025 AU, *n* = 7; novel taste 0.861 ± 0.052 AU, *n* = 7; Student’s *t* test, *t* = 2.247 df = 12, *p* = 0.0443) and inhibitory neurons (familiar taste 0.995 ± 0.087 AU, *n* = 7; novel taste 0.697 ± 0.069 AU, *n* = 7; Student’s *t* test, *t* = 2.665 df = 12, *p* = 0.0206). ***E***, QR2 mRNA FISH-labeled expression levels are not significantly reduced in layers 5/6 in the aIC in both excitatory (familiar taste 1.008 ± 0.019 AU, *n* = 7; novel taste 0.923 ± 0.061 AU, *n* = 7; Mann–Whitney test, *p* = 0.0973) and inhibitory neurons (familiar taste 1.007 ± 0.117 AU, *n* = 7; novel taste 0.764 ± 0.062 AU, *n* = 7; Student’s *t* test, *t* = 1.816 df = 12, *p* = 0.0944). ***F***, Representative image of the mouse aIC probed for vGlut (white), GAD (green), and QR2 (red). Outlines denote aIC and subregions (gIC, granular IC; dgIC, disgranular IC; agIC, agranular IC). Data are shown as mean ± SEM; *p < 0.05.

Overall, a relatively uniform reduction in QR2 expression was seen across the aIC following novel taste consumption (Fig. 2*F*), in agreement with previously published qPCR results of whole aIC homogenates (Rappaport et al., 2015; Gould et al., 2020b). Specifically, in excitatory neurons, an 8–18% reduction in QR2 expression was seen across all the different subregions and layers of the aIC (Fig. 2*A–E*, left panels). QR2 expression in excitatory neurons was most prominently and significantly reduced in layers 2/3, while also trending most strongly in the dgIC. Inhibitory neurons similarly showed a uniform reduction in QR2 expression across the aIC following a novel taste, although to a greater extent, ranging between 20% and 30% less QR2 compared with following a familiar taste. Here, significant reduction in QR2 levels were measured in layers 2/3 once more, and also in the gIC, with otherwise most strongly trending QR2 expression reduction seen also in the dgIC and layers 5/6 (Fig. 2*A–E*, right panels).

### SST interneurons are the primary locus of QR2 expression reduction in the aIC following novel taste learning

**Figure 3.**
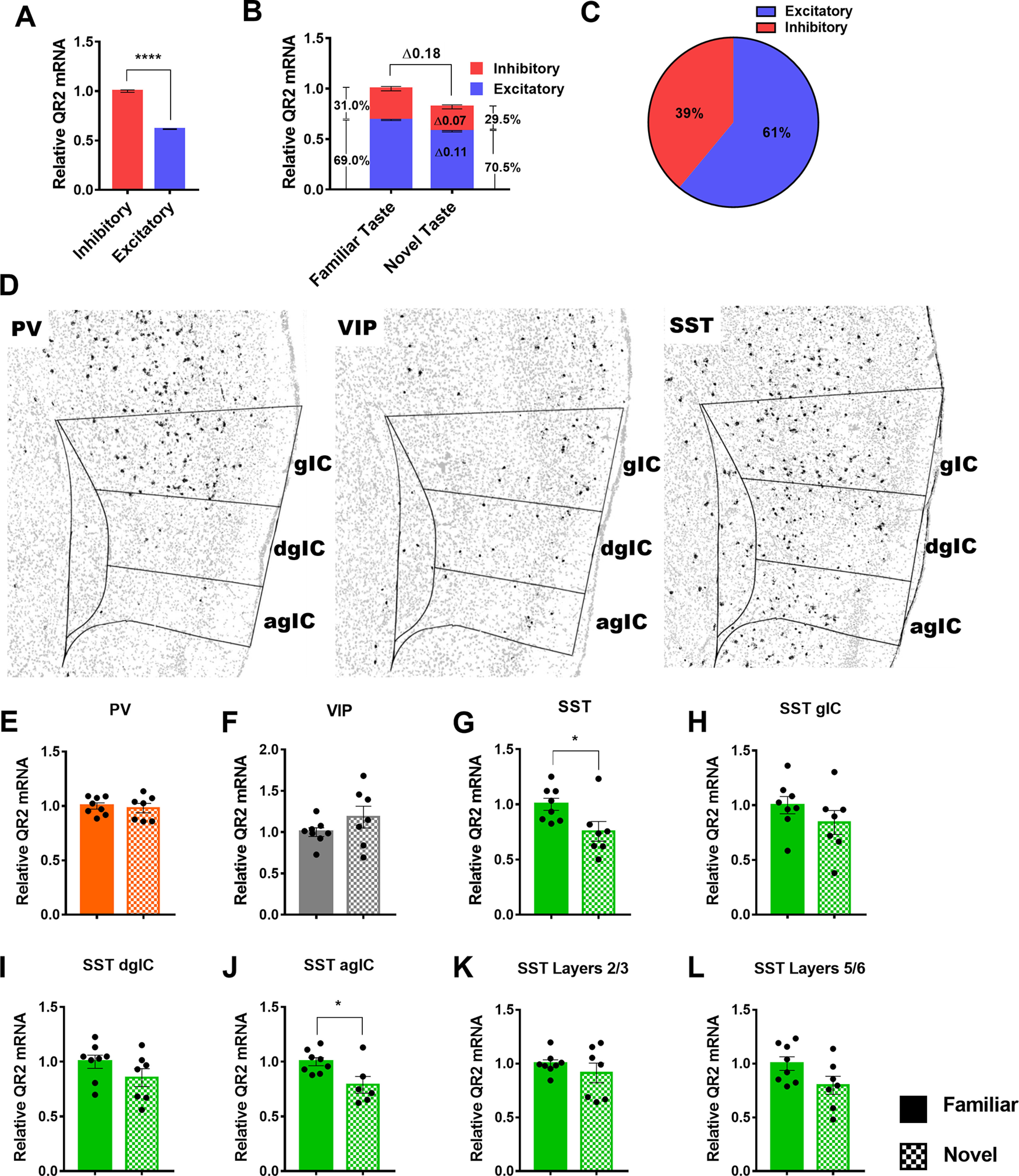
SST interneurons are the primary locus of QR2 expression reduction in the aIC following novel taste learning. ***A***, Inhibitory neurons express roughly twice as much QR2 compared with excitatory neurons (inhibitory 1 ± 0.012 AU, cells *n* = 12,592 pooled from *n* = 14 mice; excitatory 0.614 ± 0.003, cells *n* = 45,749 pooled from *n* = 14 mice; Mann–Whitney test, *p* < 0.0001). ***B***, Relative contribution of inhibitory and excitatory neurons to QR2 mRNA signal in both familiar and novel taste groups, as well as relative contribution to signal reduction following novel taste. ***C***, Percentage of the total QR2 mRNA reduction measured in the aIC, by cell type. ***D***, Representative distribution of PV, VIP, and SST interneurons in the aIC of mice, using RNAscope FISH. Outlines denote aIC and subregions (gIC, granular IC; dsIC, disgranular IC; agIC, agranular IC). ***E***, QR2 mRNA FISH-labeled expression levels are not significantly altered in PV-expressing interneurons in the aIC following novel taste consumption (PV, familiar 1 ± 0.028 AU, *n* = 8; PV, novel 0.980 ± 0.043 AU, *n* = 7; Student’s *t* test, *t* = 0.3954 df = 13, *p* = 0.6989). ***F***, QR2 mRNA FISH-labeled expression levels are not significantly altered in VIP-expressing interneurons in the aIC following novel taste consumption (VIP, familiar 1 ± 0.052 AU, *n* = 8; VIP, novel 1.182 ± 0.132 AU, *n* = 7 mice; Student’s *t* test, *t* = 1.342 df = 13, *p* = 0.2025). ***G***, QR2 mRNA FISH-labeled expression levels are reduced in SST-expressing interneurons in the aIC following novel taste consumption (SST, familiar 1 ± 0.054 AU, *n* = 8; SST, novel 0.754 ± 0.088 AU, *n* = 7; Student’s *t* test, *t* = 2.413 df = 13, *p* = 0.0313). ***H***, QR2 mRNA FISH-labeled expression levels are not significantly reduced in SST-expressing interneurons in the gIC following novel taste consumption (SST, familiar 1 ± 0.078 AU, *n* = 8; SST, novel 0.841 ± 0.109 AU, *n* = 7; Student’s *t* test, *t* = 1.198 df = 13, *p* = 0.2525). ***I***, QR2 mRNA FISH-labeled expression levels are not significantly reduced in SST-expressing interneurons in the dgIC following novel taste consumption (SST, familiar 1 ± 0.060 AU, *n* = 8; SST, novel 0.854 ± 0.082 AU, *n* = 7; Student’s *t* test, *t* = 1.456 df = 13, *p* = 0.1692). ***J***, QR2 mRNA FISH-labeled expression levels are significantly reduced in SST-expressing interneurons in the agIC following novel taste consumption (SST, familiar 1 ± 0.036 AU, *n* = 8; SST, novel 0.788 ± 0.076 AU, *n* = 6; Student’s *t* test, *t* = 2.694 df = 12, *p* = 0.0195). ***K***, QR2 mRNA FISH-labeled expression levels are not significantly reduced in SST-expressing interneurons in layers 2/3 following novel taste consumption (SST, familiar 1 ± 0.035 AU, *n* = 8; SST, novel 0.912 ± 0.090 AU, *n* = 7; Student’s *t* test, *t* = 0.9477 df = 13, *p* = 0.3606). ***L***, QR2 mRNA FISH-labeled expression levels are not significantly reduced in SST-expressing interneurons in layers 5/6 following novel taste consumption (SST, familiar 1 ± 0.062 AU, *n* = 8; SST, novel 0.797 ± 0.084 AU, *n* = 7; Student’s *t* test, *t* = 1.956 df = 13, *p* = 0.0722). Data are shown as mean ± SEM; *p < 0.05.

Next, we aimed to compare the relative QR2 levels found in excitatory and inhibitory neurons, and find the primary cell types within which QR2 expression and its suppression occur. However, distinction of single excitatory neurons is hampered in places where cell density is high and neurons overlap, thus creating difficulties for software to differentiate more than or equal to two neurons in those areas. This was not the case for the more sparsely populated, dispersed inhibitory neurons for which accurate cell count was not an issue. This resulted in a somewhat underestimated excitatory (and total) neuron cell count, and therefore a reduced ratio of excitatory-to-inhibitory neurons (∼3.6:1). However, despite this artificial reduction in total excitatory neuron number (Tremblay et al., 2016) by automated software analysis, on average inhibitory neurons were found to express roughly twice the amount of QR2 compared with excitatory neurons (Fig. 3*A*). It is therefore likely that because of the under estimation in excitatory neuron number, the ratio of excitatory to inhibitory neuronal QR2 expression in the aIC is probably even lower. Furthermore, of the total 18% reduction in QR2 expression measured 3 h following novel taste consumption within the two principal neuronal subtypes, 11% was measured in excitatory and 7% in inhibitory neurons (Fig. 3*B*). Therefore, although excitatory neurons actually outnumber inhibitory neurons by ∼7:1 in the cortex (Tremblay et al., 2016), there is a nearly 40% inhibitory to 60% excitatory neuron contribution to the overall QR2 expression reduction measured (Fig. 3*C*). This shows that QR2 is disproportionately expressed and modulated, subsequent to novel taste, within inhibitory interneurons in the aIC.

Inhibitory interneurons are heterogeneous, with distinct genetic, morphologic, physiological and functional properties (Kepecs and Fishell, 2014). We therefore probed three of the major distinct interneuron subtypes present in the aIC, namely PV, VIP, and SST (Rudy et al., 2011), which represent most (∼85%) of the interneurons in the cortex and nearly all of those located in layers 2–6 (Kepecs and Fishell, 2014; Tremblay et al., 2016). Using RNAscope FISH, we found that, as described in the literature (Kim et al., 2017), the PV-to-SST ratio in the agranular regions is much lower than in granular cortical regions (Fig. 3*D*, left and right panels), making SST the predominant interneuron in the aIC, while VIP interneurons are comparatively constrained to the superficial layers of the aIC (Fig. 3*D*, middle panel). Within these three inhibitory neuron subtypes in the whole aIC, a significant reduction in QR2 mRNA expression was seen only in SST interneurons, correlatively to novel taste consumption, while PV and VIP interneurons showed no significant changes (Fig. 3*E–G*). When analyzing aIC subregions, no changes were seen following novel taste consumption in PV or VIP interneurons in the gIC (PV, familiar 1 ± 0.026 AU, *n* = 8; PV, novel 1.013 ± 0.054 AU, *n* = 7; Student’s *t* test, *t* = 0.2208 df = 13, *p* = 0.8287; VIP, familiar 1 ± 0.062 AU, *n* = 8; VIP, novel 1.081 ± 0.175 AU, *n* = 7; Student’s *t* test, *t* = 0.4621 df = 13, *p* = 0.6517), the dgIC (PV, familiar 1 ± 0.088 AU, *n* = 7; PV, novel 0.994 ± 0.135 AU, *n* = 7; Student’s *t* test, *t* = 0.03,622 df = 12, *p* = 0.9717; VIP, familiar 1 ± 0.087 AU, *n* = 8; VIP, novel 1.255 ± 0.207 AU, *n* = 7; Student’s *t* test, *t* = 1.185 df = 13, *p* = 0.2571), the agIC (PV, familiar 1 ± 0.140 AU, *n* = 7; PV, novel 0.942 ± 0.047 AU, *n* = 7; Student’s *t* test, *t* = 0.3876 df = 12, *p* = 0.7051; VIP, familiar 1 ± 0.087 AU, *n* = 8; VIP, novel 1.255 ± 0.207 AU, *n* = 7; Student’s *t* test, *t* = 1.185 df = 13, *p* = 0.2571), layers 2/3 (PV, familiar 1 ± 0.044 AU, *n* = 8; PV, novel 1.037 ± 0.069 AU, *n* = 7; Student’s *t* test, *t* = 0.4553 df = 13, *p* = 0.6564; VIP, familiar 1 ± 0.087 AU, *n* = 8; VIP, novel 1.086 ± 0.083 AU, *n* = 7; Student’s *t* test, *t* = 0.7199 df = 13, *p* = 0.4843) or layers 5/6 (PV, familiar 1 ± 0.027 AU, *n* = 8; PV, novel 1.035 ± 0.057 AU, *n* = 7; Student’s *t* test, *t* = 0.5666 df = 13, *p* = 0.5807; VIP, familiar 1 ± 0.098 AU, *n* = 8; VIP, novel 1.558 ± 0.330 AU, *n* = 7; Student’s *t* test, *t* = 1.713 df = 13, *p* = 0.1104). In contrast, a significant reduction in QR2 was measured in SST neurons within the agIC, with strong trends seen in other regions, most notably layers 5/6 (Fig. 3*H–L*). This points to SST interneurons as the primary site of effect for the QR2 pathway, being the only major interneuron subtype assessed in the aIC within which QR2 is naturally suppressed after novel taste experience.

### QR2 suppression reduces SST interneuron excitability and increases mAHP

**Figure 4.**
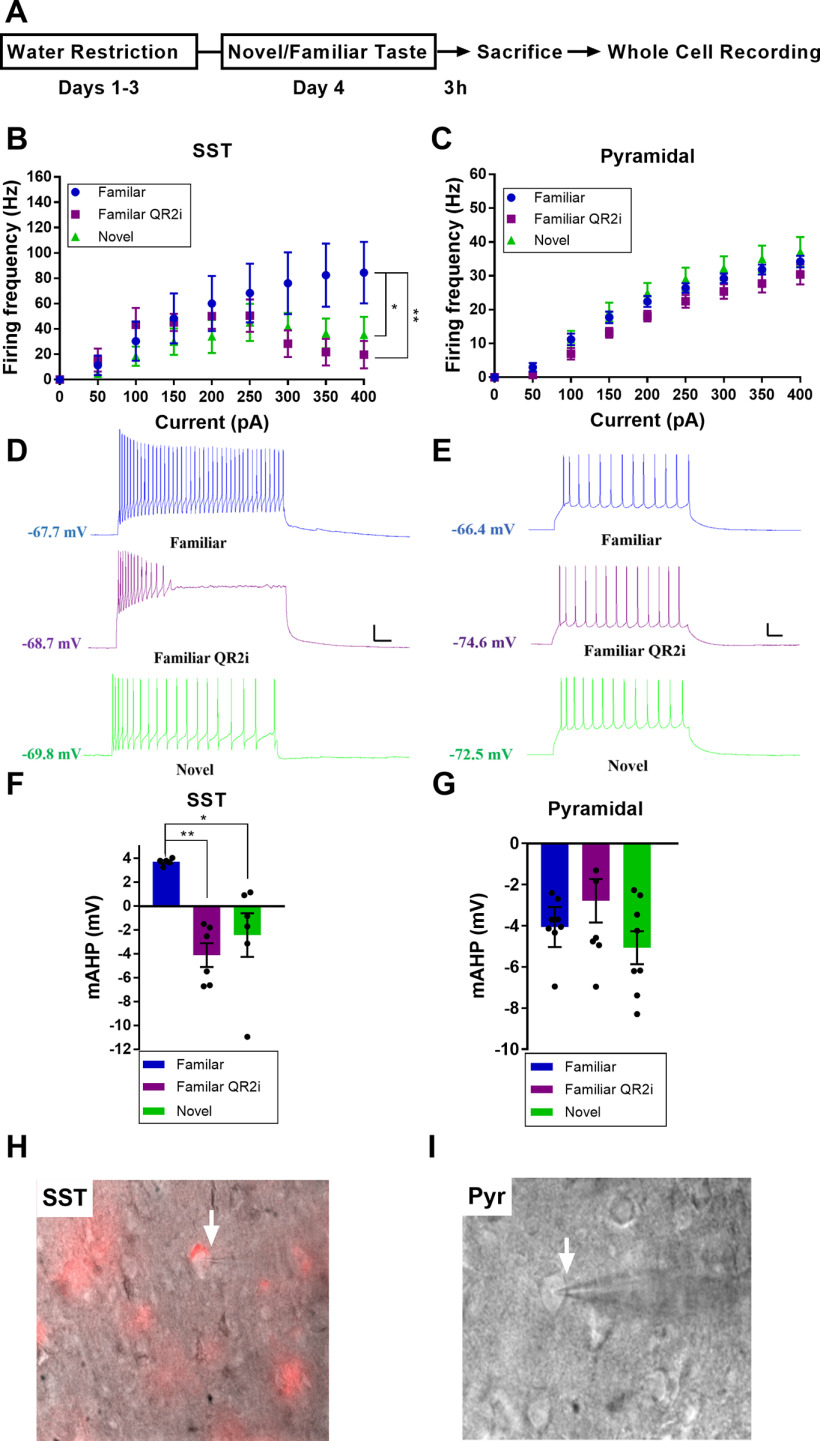
QR2 suppression reduces SST interneuron excitability and increases mAHP. ***A***, Mice were trained to drink from pipettes and were then given either a novel (saccharin) or familiar (water) taste. The mice were killed 3 h later, at a time QR2 is suppressed following novelty, and whole-cell patch recording was conducted in SST and pyramidal neurons. QR2 inhibitor (QR2i) was added into the recording pipette following familiar taste consumption, to compare with the novel taste group. ***B***, Experience of a novel taste reduces SST interneuron excitability in the aIC 3 h following consumption and QR2 inhibition 3 h following a familiar taste mimics the reduced firing frequency following novelty [two-way repeated measures ANOVA, interaction *F*_(16,120)_ = 2.707, *p* < 0.0011; pulse *F*_(8,120)_ = 12.91, *p* < 0.0001; groups *F*_(2,15)_ = 1.526, *p* = 0.2492; subjects (matching) *F*_(15,120)_ = 10.88, *p* < 0.0001; Sidak’s multiple comparisons test of familiar vs familiar QR2i at 300 pA, *p* = 0. 0.0365; familiar vs familiar QR2i at 350 pA, *p* = 0. 0.0047, familiar vs novel at 350, *p* = 0.0576; familiar vs familiar QR2i at 400, *p* = 0.0023, familiar vs novel at 400, *p* = 0.0384]. ***C***, Excitatory primary neurons of the aIC show no change in firing frequency 3 h following novel taste consumption or inhibition of QR2 [two-way repeated measures ANOVA, Interaction *F*_(16,192)_ = 1.102, *p* < 0.3555; pulse *F*_(8,192)_ = 269.8, *p* < 0.0001; groups *F*_(2,24)_ = 1.873, *p* = 0.1754; subjects (matching) *F*_(24,192)_ = 16.95, *p* < 0.0001]. ***D***, Representative traces from SST interneurons in the aIC. Scale bar: 20 mV (vertical), 50 ms (horizontal) from 350-pA current steps. ***E***, Representative traces from pyramidal neurons in the aIC. Scale bar: 20 mV (vertical), 50 ms (horizontal) from 250-pA current steps. ***F***, Experience of a novel taste increases SST interneuron mAHP in the aIC 3 h following consumption and QR2 inhibition 3 h following a familiar taste mimics the increase in mAHP following novelty (familiar 3.714 ± 0.131 mV, *n* = 5 cells from *n* = 3 mice; familiar QR2i −4.103 ± 0.995 mV, *n* = 6 cells from *n* = 3 mice; novel −2.425 ± 1.825 mV, *n* = 6 cells from *n* = 4 mice; one-way ANOVA, *F*_(2,14)_ = 9.71, *p* = 0.0023; Tukey’s multiple comparisons *post hoc* test, familiar vs familiar QR2i, *p* = 0.0022; familiar vs novel, *p* = 0.013; familiar QR2i vs novel, *p* = 0.6165). ***G***, Experience of a novel taste does not affect pyramidal neuron mAHP in the aIC 3 h following consumption and QR2 inhibition following a familiar taste has no effect on mAHP (familiar −4.063 ± 0.972 mV, *n* = 10 cells from *n* = 3 mice; familiar QR2i −2.788 ± 1.053 mV, *n* = 8 cells from *n* = 3 mice; novel −5.066 ± 0.799 mV, *n* = 8 cells from *n* = 3 mice; one-way ANOVA, *F*_(2,23)_ = 1.312, *p* = 0.2887). ***H***, Representative image of mCherry-expressing SST interneuron in the aIC. ***I***, Representative image of pyramidal neuron in the aIC. Data are shown as mean ± SEM; *p < 0.05, **p < 0.005.

**Figure 5.**
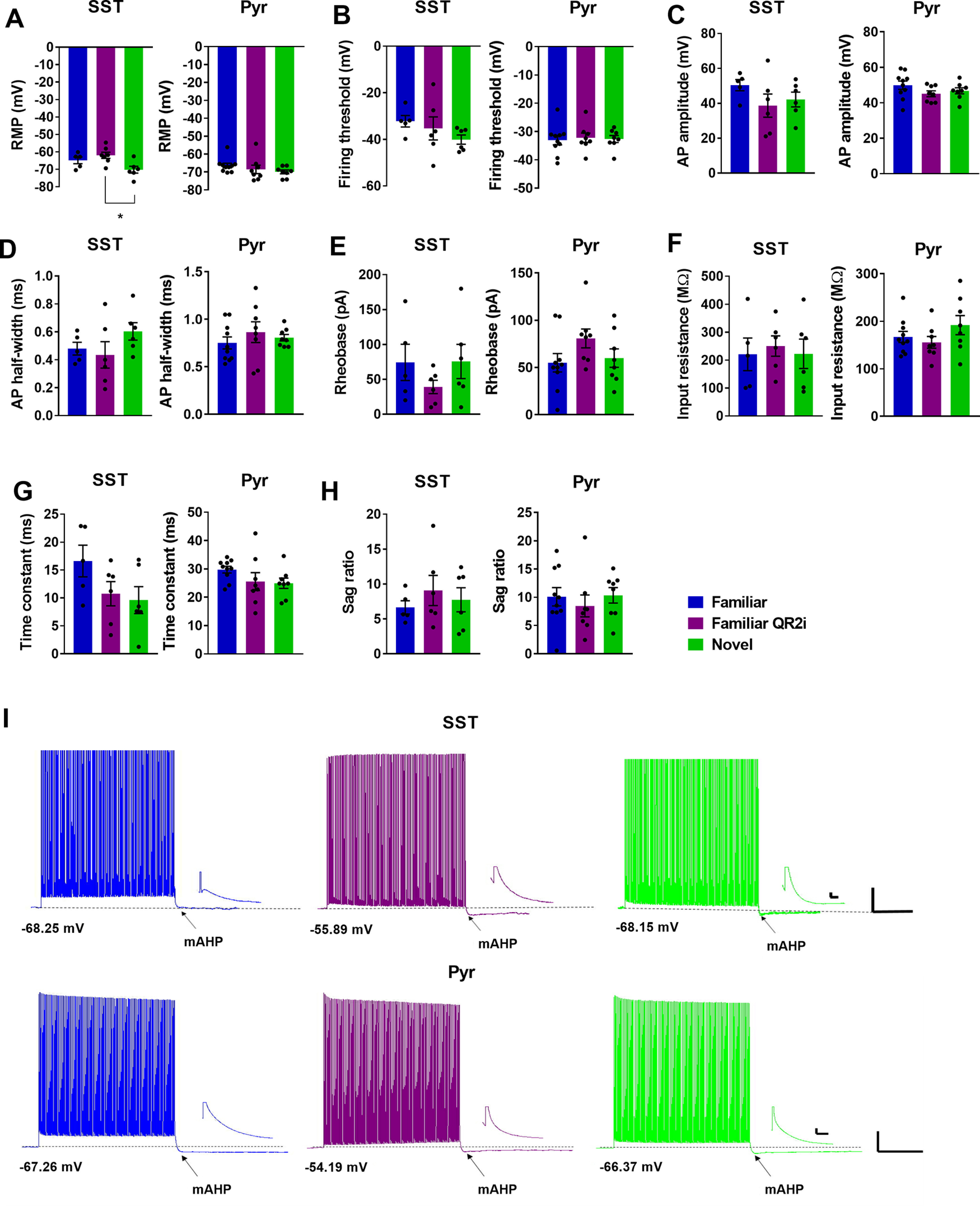
QR2 inhibition alters intrinsic properties of SST but not pyramidal neurons in the aIC. ***A***, RMP of SST interneurons of the aIC is hyperpolarized following novel taste experience compared with familiar taste with QR2 inhibition, but not when compared with familiar taste alone (familiar −64.85 ± 1.924 mV, *n* = 5 cells from *n* = 3 mice; familiar QR2i −61.96 ± 1.177 mV, *n* = 7 cells from *n* = 3 mice; novel −70.21 ± 1.948 mV, *n* = 6 cells from *n* = 4 mice; one-way ANOVA, *F*_(2,15)_ = 5.22, *p* = 0.019; Tukey’s multiple comparisons *post hoc* test, familiar vs familiar QR2i, *p* = 0.5471; familiar vs novel, *p* = 0.1687; familiar QR2i vs novel, *p* = 0.0152). RMP of pyramidal neurons of the aIC remains unchanged 3 h following novel taste experience or QR2 inhibition (familiar −66.39 ± 1.271 mV, *n* = 10 cells from *n* = 3 mice; familiar QR2i −68.54 ± 2.336 mV, *n* = 8 cells from *n* = 3 mice; novel −69.93 ± 1.068 mV, *n* = 8 cells from *n* = 3 mice; Kruskal–Wallis test, *p* = 0.1301). ***B***, Firing threshold of SST interneurons in the aIC remains unchanged 3 h following novel taste experience or QR2 inhibition (familiar −32.21 ± 2.476 mV, *n* = 5 cells from *n* = 3 mice; familiar QR2i −35.31 ± 4.946 mV, *n* = 6 cells from *n* = 3 mice; novel −40.13 ± 1.965 mV, *n* = 6 cells from *n* = 4 mice; one-way ANOVA, *F*_(2,14)_ = 1.274, *p* = 0.3103). Firing threshold of pyramidal neurons in the aIC remains unchanged 3 h following novel taste experience or QR2 inhibition (familiar −33.05 ± 1.647 mV, *n* = 10 cells from *n* = 3 mice; familiar QR2i −32.19 ± 1.657 mV, *n* = 8 cells from *n* = 3 mice; novel −32.64 ± 1.218 mV, *n* = 8 cells from *n* = 3 mice; one-way ANOVA, *F*_(2,23)_ = 0.07,867, *p* = 0.9246). ***C***, Action potential amplitude of SST interneurons in the aIC remains unchanged 3 h following novel taste experience or QR2 inhibition (familiar 50.4 ± 3.183 mV, *n* = 5 cells from *n* = 3 mice; familiar QR2i 38.66 ± 6.677 mV, *n* = 6 cells from *n* = 3 mice; novel 42.17 ± 4.206 mV, *n* = 6 cells from *n* = 4 mice; one-way ANOVA, *F*_(2,14)_ = 1.316, *p* = 0.2993). Action potential amplitude of pyramidal neurons in the aIC remains unchanged 3 h following novel taste experience or QR2 inhibition (familiar 49.91 ± 2.328 mV, *n* = 10 cells from *n* = 3 mice; familiar QR2i 45.11 ± 1.616 mV, *n* = 8 cells from *n* = 3 mice; novel 46.74 ± 1.702 mV, *n* = 8 cells from *n* = 3 mice; one-way ANOVA, *F*_(2,23)_ = 1.562, *p* = 0.2311). ***D***, Action potential half width of SST interneurons in the aIC remains unchanged 3 h following novel taste experience or QR2 inhibition (familiar 0.48 ± 0.045 ms, *n* = 5 cells from *n* = 3 mice; familiar QR2i 0.435 ± 0.094 ms, *n* = 6 cells from *n* = 3 mice; novel 0.603 ± 0.062 mV, *n* = 6 cells from *n* = 4 mice; one-way ANOVA, *F*_(2,14)_ = 1.474, *p* = 0.2624). Action potential half width of pyramidal neurons in the aIC remains unchanged 3 h following novel taste experience or QR2 inhibition (familiar 0.751 ± 0.063 ms, *n* = 10 cells from *n* = 3 mice; familiar QR2i 0.863 ± 0.109 ms, *n* = 8 cells from *n* = 3 mice; novel 0.806 ± 0.034 mV, *n* = 8 cells from *n* = 3 mice; one-way ANOVA, *F*_(2,23)_ = 0.597, *p* = 0.5588). ***E***, Rheobase of SST interneurons in the aIC remains unchanged 3 h following novel taste experience or QR2 inhibition (familiar 74.4 ± 25.97 pA, *n* = 5 cells from *n* = 3 mice; familiar QR2i 39.17 ± 9.61 pA, *n* = 6 cells from *n* = 3 mice; novel 75.67 ± 24.55 pA, *n* = 6 cells from *n* = 4 mice; one-way ANOVA, *F*_(2,14)_ = 1.022, *p* = 0.3851). Rheobase of pyramidal neurons in the aIC remains unchanged 3 h following novel taste experience or QR2 inhibition (familiar 55.00 ± 9.698 pA, *n* = 10 cells from *n* = 3 mice; familiar QR2i 80.75 ± 10.03 pA, *n* = 8 cells from *n* = 3 mice; novel 59.88 ± 9.781 pA, *n* = 8 cells from *n* = 3 mice; one-way ANOVA, *F*_(2,23)_ = 1.884, *p* = 0.1747). ***F***, Input resistance of SST interneurons in the aIC remains unchanged 3 h following novel taste experience or QR2 inhibition (familiar 220.8 ± 58.51 MΩ, *n* = 5 cells from *n* = 3 mice; familiar QR2i 250.3 ± 36.47 MΩ, *n* = 6 cells from *n* = 3 mice; novel 222.5 ± 52.49 MΩ, *n* = 6 cells from *n* = 4 mice; one-way ANOVA, *F*_(2,14)_ = 0.1159, *p* = 0.8914). Input resistance of pyramidal neurons in the aIC remains unchanged 3 h following novel taste experience or QR2 inhibition (familiar 167.1 ± 11.67 MΩ, *n* = 10 cells from *n* = 3 mice; familiar QR2i 155.5 ± 12.38 MΩ, *n* = 8 cells from *n* = 3 mice; novel 191.9 ± 20.18 MΩ, *n* = 8 cells from *n* = 3 mice; one-way ANOVA, *F*_(2,23)_ = 1.466, *p* = 0.2517). ***G***, Time constant of SST interneurons in the aIC remains unchanged 3 h following novel taste experience or QR2 inhibition (familiar 16.63 ± 2.828 ms, *n* = 5 cells from *n* = 3 mice; familiar QR2i 10.76 ± 2.169 ms, *n* = 6 cells from *n* = 3 mice; novel 9.619 ± 2.397 ms, *n* = 6 cells from *n* = 4 mice; one-way ANOVA, *F*_(2,14)_ = 2.219, *p* = 0.1455). Time constant of pyramidal neurons in the aIC remains unchanged 3 h following novel taste experience or QR2 inhibition (familiar 29.73 ± 1.177 ms, *n* = 10 cells from *n* = 3 mice; familiar QR2i 25.52 ± 3.137 ms, *n* = 8 cells from *n* = 3 mice; novel 24.93 ± 1.837 ms, *n* = 8 cells from *n* = 3 mice; one-way ANOVA, *F*_(2,23)_ = 1.677, *p* = 0.2090). ***H***, Sag ratio of SST interneurons in the aIC remains unchanged 3 h following novel taste experience or QR2 inhibition (familiar 6.647 ± 0.950, *n* = 5 cells from *n* = 3 mice; familiar QR2i 9.08 ± 2.158, *n* = 6 cells from *n* = 3 mice; novel 7.742 ± 1.724, *n* = 6 cells from *n* = 4 mice; one-way ANOVA, *F*_(2,14)_ = 0.4634, *p* = 0.6384). Sag ratio of pyramidal neurons in the aIC remains unchanged 3 h following novel taste experience or QR2 inhibition (familiar 10.09 ± 1.621, *n* = 10 cells from *n* = 3 mice; familiar QR2i 8.454 ± 1.934, *n* = 8 cells from *n* = 3 mice; novel 10.33 ± 1.376, *n* = 8 cells from *n* = 3 mice; one-way ANOVA, *F*_(2,23)_ = 0.3548, *p* = 0.7051). ***I***, Representative mAHP traces from SST and pyramidal neurons in the aIC. Scale bars: 20 mV and 1 s (large) and 5 mv and 20 ms (small). Dashed line indicates baseline RMP. Data are shown as mean ± SEM; *p < 0.05.

Following the cell type profile of QR2 suppression seen in the aIC 3 h subsequent to novel taste consumption, we aimed to determine what effect reduced QR2 expression has on the cell types identified, namely SST interneurons and excitatory pyramidal neurons. To do so, SST-Cre mice (The Jackson Laboratory stock #013044) injected with a Cre-dependent mCherry reporter, or wild-type (WT) mice, were given a familiar (water) or novel (saccharin 0.5%) taste to drink. The mice were then killed 3 h later to perform whole-cell patch-clamp recordings of the intrinsic properties of SST or pyramidal neurons of the aIC (Fig. 4*A*). Interestingly, 3 h following novel taste consumption, SST but not pyramidal neurons showed reduced firing frequency (Fig. 4*B–E*). Remarkably, by inhibiting QR2 in neurons of animals that drank a familiar taste (water), using 0.5 μm S29434 (Ferry et al., 2010) within the recording pipette, the firing frequency of aIC SST (Fig. 4*H*) but not pyramidal (Fig. 4*I*) neurons was reduced similarly, if more strongly, to that seen following novel taste (Fig. 4*B–E*). Additionally, the mAHP of SST but not pyramidal neurons was increased following novel taste consumption (Fig. 5*I*), and QR2 inhibition within SST neurons of animals that experienced a familiar taste mimicked the novel taste mediated effect (Fig. 4*F*,*G*). Other intrinsic properties measured were not significantly affected by novel taste consumption alone in either SST or excitatory neurons (Fig. 5*A–H*). However, QR2 inhibition in SST neurons of mice experiencing a familiar taste resulted in significantly more hyperpolarized RMP compared with SST neurons of mice experiencing a novel taste (Fig. 5*A*). Together, this provides both a cellular pattern and function for the QR2 pathway within the circuits of the aIC in novel taste learning.

### Reduced QR2 expression in SST interneurons enhances novel taste memory formation

**Figure 6.**
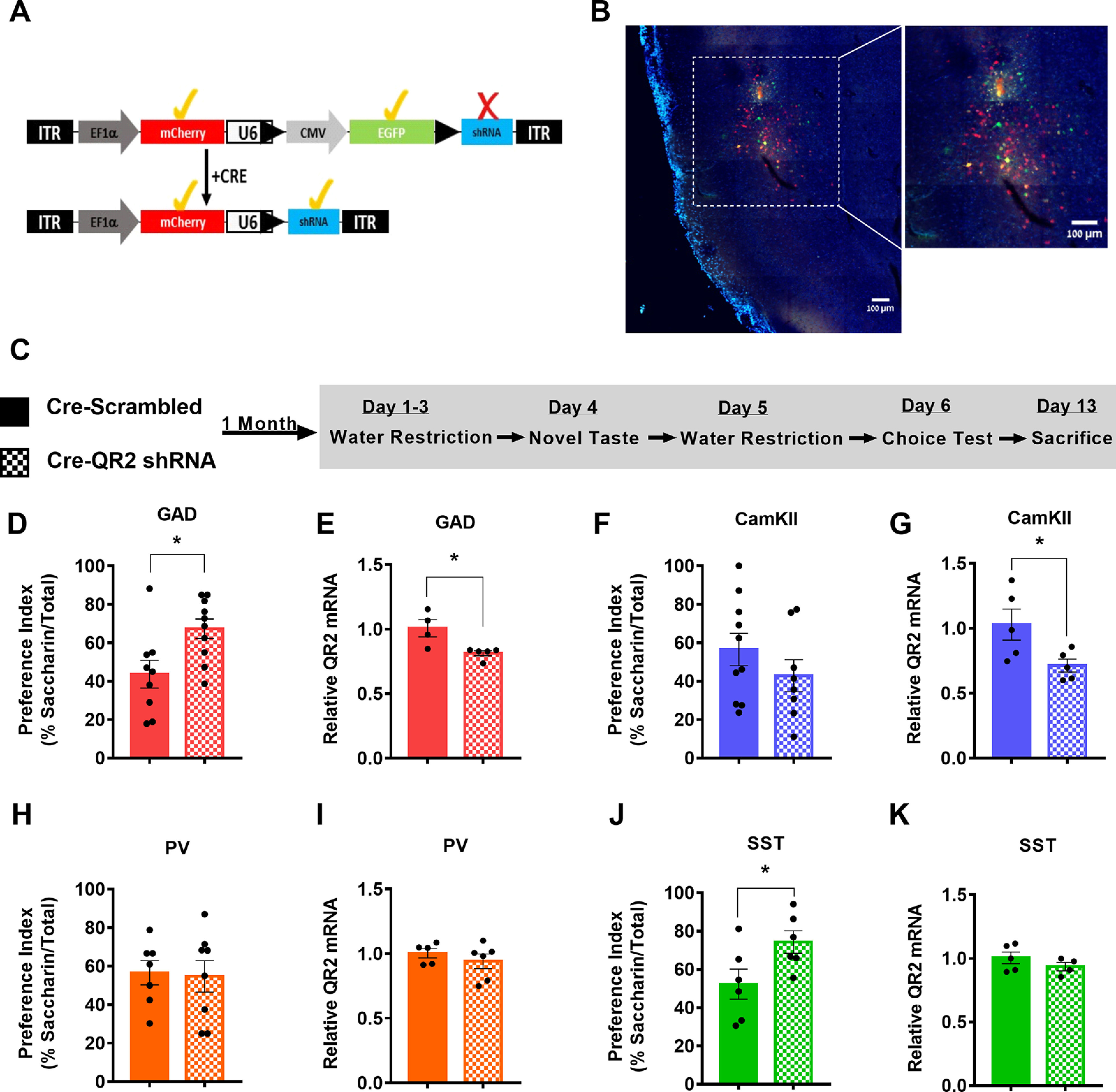
Reduced QR2 expression in SST interneurons enhances novel taste memory formation. ***A***, Schematic diagram of pAAV-Sico-Red-QR2 shRNA vector before and after Cre-mediated recombination. ***B***, Representative image of Cre-dependent QR2 shRNA viral vector expression in GAD Cre mouse aIC. ***C***, Mice were injected with the Cre-dependent QR2 shRNA or scrambled viral vector to the aIC. A month later, they underwent novel taste learning, and 2 d after consuming the novel taste, they were given a choice test and a preference index was made. A week later, the mice were killed to assess infection efficacy, and the effect the expression of the virus had on local QR2 mRNA expression. ***D***, GAD Cre mice injected with Cre-dependent QR2 shRNA showed significantly improved novel taste memory compared with scrambled controls (GAD Cre-Scrambled 43.68 ± 7.218%, *n* = 9; GAD Cre-QR2 shRNA 67.31 ± 5.096%; *n* = 10; Student’s *t* test, *t* = 2.717 df = 17, *p* = 0.0146). ***E***, GAD Cre mice injected with Cre-dependent QR2 shRNA showed significantly reduced QR2 mRNA expression compared with scrambled controls (GAD Cre-Scrambled 1.007 ± 0.066 2^-ΔΔCt^, *n* = 4; GAD Cre-QR2 shRNA 0.813 ± 0.019 2^-ΔΔCt^, *n* = 5; Mann–Whitney test, *p* = 0.0159). ***F***, Mice co-expressing Cre under the CamKII promoter and Cre-dependent QR2 shRNA in the aIC show no significant change in novel taste memory compared with those injected with the scrambled Cre-dependent virus (CamKII Cre-Scrambled 56.48 ± 8.423%, *n* = 10; CamKII Cre-QR2 shRNA 42.84 ± 8.324%, *n* = 8; Student’s *t* test, *t* = 1.136 df = 16, *p* = 0.2728). ***G***, A significant reduction in QR2 mRNA was measured in mice co-expressing Cre under CamKII promoter and Cre-dependent QR2 shRNA in the aIC, compared with controls (CamKII Cre-Scrambled 1.027 ± 0.119 2^-ΔΔCt^, *n* = 5; CamKII Cre-QR2 shRNA 0.7122 ± 0.049 2^-ΔΔCt^, *n* = 5; Student’s *t* test, *t* = 2.435 df = 8, *p* = 0.0409). ***H***, PV Cre mice injected with Cre-dependent QR2 shRNA in the aIC show no significant change in novel taste memory compared with those injected with the scrambled Cre-dependent virus (PV Cre-Scrambled 56.56 ± 6.274%, *n* = 7; PV Cre-QR2 shRNA 54.67 ± 8.180%, *n* = 8; Student’s *t* test, *t* = 0.1792 df = 13, *p* = 0.8605). ***I***, PV Cre mice injected with Cre-dependent QR2 shRNA showed insignificant QR2 mRNA expression reduction compared with scrambled controls (PV Cre-Scrambled 1.003 ± 0.035 2^-ΔΔCt^, *n* = 5; PV Cre-QR2 shRNA 0.940 ± 0.056 2^-ΔΔCt^, *n* = 6; Student’s *t* test, *t* = 0.8789 df = 9, *p* = 0.4023). ***J***, SST Cre mice injected with Cre-dependent QR2 shRNA showed significantly improved novel taste memory compared with scrambled controls (SST Cre-Scrambled 52.26 ± 7.868%, *n* = 6; SST Cre-QR2 shRNA 74.26 ± 5.843%; *n* = 6; Student’s *t* test, *t* = 2.246 df = 10, *p* = 0.0485). ***K***, SST Cre mice injected with Cre-dependent QR2 shRNA showed insignificant QR2 mRNA expression reduction compared with scrambled controls (SST Cre-Scrambled 1.004 ± 0.046 2^-ΔΔCt^, *n* = 5; SST Cre-QR2 shRNA 0.935 ± 0.032 2^-ΔΔCt^, *n* = 4; Student’s *t* test, *t* = 1.154 df = 7, *p* = 0.2864). Data are shown as mean ± SEM; *p < 0.05.

To investigate whether the cellular pattern of reduced QR2 expression could also indicate QR2 mediated effect on memory and behavior, we used selective QR2 suppression in inhibitory or excitatory neurons of the aIC. To do so, we generated an adeno associated viral vector containing the pSico-Red (Jung et al., 2016) construct to target QR2 mRNA for degradation on Cre recombinase activation (Fig. 6*A*). This was injected into the aIC of GAD-Cre transgenic mice (The Jackson Laboratory stock #010802; Fig. 6*B*), or WT mice in tandem with another viral vector, expressing Cre under the CamKII promoter (see Materials and Methods). Mice were given a month to recover and then trained to drink from pipettes and consume a novel taste (saccharin), for which their memory was tested 48 h following learning, using a choice test (Fig. 6*C*). Mice expressing QR2 shRNA in inhibitory neurons (mCherry reporter expression in GAD Cre-Scrambled 9.006 ± 0.084 ΔC_t_, GAD Cre-QR2 shRNA 9.306 ± 0.032 ΔC_t_) showed both significantly reduced QR2 expression (Fig. 6*E*) and improved memory compared with controls (Fig. 6*D*). In contrast, mice with QR2 shRNA targeted to excitatory neurons (mCherry reporter expression in CamKII Cre-Scrambled 8.345 ± 0.150 ΔC_t_, CamKII Cre-QR2 shRNA 8.849 ± 0.089 ΔC_t_) showed no improvement in novel taste memory (Fig. 6*F*), although a significant reduction in QR2 was measured (Fig. 6*G*). Following this result, we aimed to determine whether SST interneurons represent the major interneuron subtype contributing to the improvement in memory observed on selective QR2 degradation in inhibitory cells, in line with the intrinsic properties measured and *in situ* results obtained. Therefore, SST-Cre and PV-Cre animals (The Jackson Laboratory stock #013044 and #008069, respectively) were injected with Cre-dependent QR2 shRNA-expressing viral vectors. In PV Cre animals (mCherry reporter expression in PV Cre-Scrambled 11.798 ± 0.711 ΔC_t_, PV Cre-QR2 shRNA 11.804 ± 0.492 ΔC_t_) a ∼6% (Fig. 6*I*) and in SST Cre animals (mCherry reporter expression in SST Cre-Scrambled 10.939 ± 0.326 ΔC_t_, SST Cre-QR2 shRNA 11.346 ± 0.783 ΔC_t_) an ∼7% (Fig. 6*K*) non-significant reduction in total aIC QR2 expression was measured by qPCR. These relatively small reductions to total aIC QR2 expression are indicative of the relatively small number of cells SST and PV interneurons represent (between 2% and 6%) from the total cell population measured in this way, within the vast majority of which (between 94% and 98%) QR2 was not targeted. In agreement with the *in situ* results (Fig. 3), QR2 shRNA expression in SST interneurons resulted in significantly better memory for the novel taste compared with controls (Fig. 6*J*), while no effect was seen in PV Cre animals (Fig. 6*H*). Combined, these results demonstrate that SST interneurons are the key locus of QR2 expression reduction involved in the consolidation of novel taste memory in the aIC.

## Discussion

Learning is a process defined by time, in which animals first acquire the information and later consolidate it molecularly (hours) and systemically (days to months; McGaugh, 2000; Squire et al., 2015b). Synaptic plasticity and molecular memory consolidation are defined by sensitivity to protein synthesis inhibitors and the involvement of specific mRNA translation factors such as eIF2α (Davis and Squire, 1984; Costa-Mattioli et al., 2007). Interestingly, we have recently shown that eIF2α mediated synaptic plasticity and memory consolidation in the hippocampus occurs in SST interneurons, as well as excitatory neurons (Sharma et al., 2020). In parallel, we described the QR2 pathway, which is a central molecular mediator of novel memory consolidation in the aIC and hippocampus, in both taste and contextual modalities (Rappaport et al., 2015; Gould et al., 2020a,b). Activation of the pathway reduces QR2 expression, which alters the redox state of neurons, among other things, by reducing physiological ROS. In the hippocampus, we found this occurred mostly in interneurons, where initially high levels of QR2 are most notably reduced. In interneurons alone, inhibition of QR2 reduces excitability, pointing to QR2 mediated redox modulation of hippocampal interneuron intrinsic properties 3 h following learning (Gould et al., 2020a). Since QR2 thus potentially alters circuit states via interneuron activity to enhance the encoding of an internal representation for a novel event in the hippocampus, we aimed to identify how this pathway is activated in the aIC, to help the relevant circuits for long-term novel memory formation there. The aIC has laminated and stratified structures, within which reside diverse cell types (Gogolla, 2017; Kim et al., 2017). Here too, we showed that 3 h following a novel experience, QR2 is reduced from high baseline levels predominantly in interneurons, similarly to CA1, and that this occurs across this entire complex cortical structure, to varying degrees. Furthermore, of the three major interneuron subtypes assessed in the aIC, QR2 expression is suppressed only within SST interneurons. Interestingly, however, the subregions within which QR2 expression reduction was seen in SST neurons does not entirely match that seen when taking the total inhibitory interneuron population. Namely, layers 2/3, in which a significant reduction in QR2 was measured across all inhibitory neurons did not show similarly reduced levels in SST cells, pointing to perhaps another interneuron subtype which may play a role via the QR2 pathway. A possible candidate inhibitory cell subtype could be neuron-derived neurotrophic factor (NDNF) interneurons, which have recently been described as a major subtype largely found in the superficial layers, and involved in brain state changes, as has been seen with QR2 elsewhere (Abs et al., 2018; Gould et al., 2020a; Cohen-Kashi Malina et al., 2021). Overall, a pattern of QR2 suppression in SST, and to a more modest extent excitatory neurons was found here. When comparing the effect on intrinsic properties that novel taste mediated QR2 suppression has on these two major cell types that contribute to the total observed reduction in QR2 expression, a stark difference emerges. At 3 h following novel taste, SST interneurons display attenuated intrinsic excitability, as well as an increase in mAHP, unlike excitatory neurons which remain unchanged compared with familiar taste at this time point. Inhibition of QR2 within SST interneurons, but not excitatory neurons of mice that drank a familiar taste alters these intrinsic properties similarly to novel taste, if to a far greater extent. This is likely indicative of the powerful pharmacological intervention (Ferry et al., 2010), and may also explain how QR2 inhibition using small molecules is able to improve normal, as well as impaired memory formation (Rappaport et al., 2015; Gould et al., 2020b). There is also an interesting twist, since although the net effect on inhibitory neurons both in the hippocampus and aIC following QR2 suppression is a reduction in firing rate, the way this occurs is different (Fig. 3; Gould et al., 2020b). A possible explanation is that these two brain regions have distinct interneuronal profiles (Pelkey et al., 2017), but also it may be because of the fact that in the hippocampus this was measured across all inhibitory neurons, whereas presently in the aIC, it is measured specifically within SST interneurons. Excitatory neurons, on the other hand, show no such novelty or QR2 inhibition mediated effects at this time point, 3 h following the experience, which represents the optimal window in which to measure QR2 expression reduction. This may be because of the fact that SST interneurons express far higher levels of QR2 to begin with, so that the proportion of QR2 removed from within these cells is far greater than that in excitatory neurons. Another option is that the distinct membrane channel profile of SST interneurons is particularly sensitive to QR2 activity, and thus strongly affects SST intrinsic properties in a manner that is unique to these cells (Huntley et al., 2020). Indeed, establishing which ion channel/s are affected by QR2 and its inhibition remain an open question, and subject for further investigation.

An intriguing possibility lies in the concept of a second wave of proteostasis rearrangement, occurring ≥3 h following learning, proposed last century by Hansjurgen Matthies (Grecksch and Matthies, 1980; Frey et al., 1988), and later by others (Bekinschtein et al., 2007; Martínez-Moreno, 2011). This second phase of molecular mechanisms underlying memory consolidation may result in different protein synthesis, depending on cell type, and may cause either QR2 activity or protein-protein interaction mediated shifts that are unique to SST interneurons. Indeed, this may equally be true for excitatory cells, where the slight decrease in QR2 observed may augment or affect longer term aspects of cell function, possibly via proteostasis (Olshina et al., 2020) for example, to stabilize the memory trace over longer time periods, differently from, but along with, SST interneurons. However, in terms of the measurable behavioral outcome of incidental taste learning, targeted removal of QR2 within inhibitory, but not excitatory neurons, enhances memory formation. Moreover, it is within SST interneurons that genetic suppression of QR2 enables enhanced novel taste learning. This further corroborates and qualifies the cellular distinction found when assessing cellular expression and intrinsic property changes following QR2 pathway activation. In contrast, PV or VIP interneuron subtypes in the aIC did not show changes in QR2 levels, and targeted QR2 suppression in PV interneurons did not affect taste memory. This may indicate that, in the present modality, novelty is represented as a cortical-state dictated by SST interneurons at the late stage of novel taste experience (Urban-Ciecko and Barth, 2016; Fuchs et al., 2017; Cardin, 2019).

The QR2 pathway has an intercellular and intracellular affect. The former is by reducing the firing rate of interneurons, thus transiently altering E/I balance. The latter is redox modulation, which may have any number of molecular targets (Choi and Lipton, 2000; Reybier et al., 2011; Bogeski and Niemeyer, 2014; Gamper and Ooi, 2015; Lefaki et al., 2017; Lorenzen et al., 2017; Sies, 2017; Naseri Kouzehgarani et al., 2020). We have previously identified Kv2.1 as one such important target, which may explain the alterations in cell excitability we see presently (Cotella et al., 2012; Gould et al., 2020b). We expect other targets besides Kv2.1 to be identified in the future, and these may be cell type specific in their expression pattern, similarly to QR2 and eIF2α. Certainly, how SST interneurons are affected by QR2 redox and via which channel or cellular component, or indeed whether QR2 pathway activation causes other, non-redox mediated changes, are additional issues that require future efforts to decipher (Olshina et al., 2020; Sonavane et al., 2020; Hayat et al., 2021). Ultimately, how this altered SST interneuron activity subsequently affects the neuronal circuit/s involved in novel taste memory formation poses a substantial and important task for future research. To wit, the aIC is not the only brain structure involved in novel taste learning, and future studies will have to integrate the local circuit within the aIC, including the newly identified SST/QR2 component, as well as other saliency/novelty hubs such as the medial prefrontal cortex.

Here, as well as in recent findings, SST interneurons have emerged as an important focal point in which it is necessary to identify both molecular and circuit wide mechanisms underlying memory consolidation. The QR2 pathway is one such mechanism that must be further investigated in these cells, as it is the molecular determinant by which SST interneurons galvanize the distinct memory that is formed to differentiate novel from familiar.

## Synthesis

Reviewing Editor: Isabel Perez-Otaño, Instituto de Neurociencias Decisions are customarily a result of the Reviewing Editor and the peer reviewers coming together and discussing their recommendations until a consensus is reached. When revisions are invited, a fact-based synthesis statement explaining their decision and outlining what is needed to prepare a revision will be listed below. The following reviewer(s) agreed to reveal their identity: Luis Garcia-Rabaneda. Note: If this manuscript was transferred from JNeurosci and a decision was made to accept the manuscript without peer review, a brief statement to this effect will instead be what is listed below.

Your manuscript has been seen by 1 reviewer who thought your work advances our current knowledge of mechanisms underlying taste learning in the insular cortex. However, she/he had suggesitons for improvement including a better illustration of the ISH data, both images and graphs.

Reviewer´s comments:

In this study, Gould et al show the cellular mechanism behind the novel taste learning in the mouse insular cortex, molecular mechanism which has been previously studied and published in two different manuscripts (Gould et al, eNeuro 2020; and Goud et al, J Neurosci 2020). In the previous manuscripts they described the role of QR2 (quinine reductase 2) during memory consolidation of the taste learning in the insular cortex (which occurs downstream of the activation of ACh receptors), and the hippocampus (through a dopamine-dependent redox modulation, which changes the intrinsic properties of inhibitory neurons).

In this manuscript, the authors try to determine which neuronal cell within the insular cortex, and which intrinsic properties, change by the reduction of QR2, after novel taste learning. To analyze the expression of QR2, they compare the RNA levels in excitatory and inhibitory cells using “RNAscope in situ hybridation”. They find decreased expression of QR2, mostly in inhibitory neurons, specifically somatostatin cells. Knowing that QR2 may affect the intrinsic properties of interneurons, they studied those properties using SST transgenic mice, and confirmed the effects in the excitability of these interneurons. Importantly, the authors investigate this specificity by downregulating the expression of QR2 in adult transgenic mice of inhibitory and excitatory cells, using to AAVs-shRNA. The authors report the phenotype in GAD-cre, and SST-Cre transgenic mice although the relative expression of QR2 did not decrease in those transgenic mice. Taken together all the results, I will be positive to publish the manuscript in eNeuro, but there are some points to improve before publishing the manuscript.

- As a general comment to the authors, the structure of the results is too difficult to read, bringing some sentences from previous reviews, which mislead the meaning of the different paragraphs. In addition, the manuscript loses the title structure to differentiate the main message of the paragraph, doing difficult to emphasize the results associated to the figures. I will recommend to the authors rewriting the results of the manuscript, establishing some structural organization.

- As Figure 1 and 2 highlight the same information, I will consider fusing in only one figure, being important to show the specific images of QR2 ISH for each layer. Besides, of the graphical results, I will recommend showing the images indicating the reduction specifically in the inhibitory neurons. As the authors have the information about the cortex (as a negative control), it will be informative to show the results in inhibitory and excitatory neurons in somatosensory cortex.

- In addition to the localization of PV, VIP and SST, please add the results of relative QR2 expression in the different cells in each layer. This fact could be interesting to link with the lack of reduction in the QR2 expression in SST-cre mice. Could it be due to the place of injection? Or the insular area where the AAV is expressed?.

- The authors should correct the part of the text link to the figures 4 to 6. Also, they should define some acronyms in the text (for example, mAHP), and they should consider fusing figures five and six in only one figure, as it is not so important for the meaning of the manuscript.

- The last figure will improve if the authors show the layering of the insular cortex in the different transgenic mice treated. It would be nice to divide this figure in two different main figures with the graphs and images associated. One showing GAD and CAMKII mice, and other PV and SST mice) to highlight the results (these figures might include the representative image expression)

## Author Response

Reviewer #1 (Rationale for Significance Rating for Authors (Required)):

The contribution of intrinsic excitability changes to taste learning is conceptually interesting, particularly

given the cell-specificity. This may have implications for other systems. However, it is unclear to me

whether this normally contributes to learning, because in the behavioral experiments (4B and J) the

scrambled shRNA groups appear to exhibit no taste preference and so whether normal physiological

QR2 changes are involved in learning is still an open question.

Reviewer 1 finds the present manuscript interesting, in particular with regards to the cell-specific nature

of the findings, describing QR2 as an important removable constraint for memory formation and posits

that the new advances described here could be implicated in other brain systems. The reviewer adds

that the experiments are well designed and the findings are novel. However, the reviewer has concerns

regarding the levels of novel taste consumption seen as a measurable metric for learning and memory.

The reviewer also questions whether normal physiological changes to QR2 expression are involved in

learning, since we did not show in the present study that high levels of QR2 prevent taste learning.

Finally, the reviewer was not able to access important data relating to the electrophysiological

experiments carried out, and was unable to assess these experiments to their satisfaction. We have

addressed each of the reviewer’s concerns in detail, both here and within the revised manuscript. The

manuscript has greatly benefitted as a result of this reviewers input, and for this we would like to thank

reviewer 1.

Reviewer #1 :

Gould et al. investigated the molecular mechanisms of long-term memory consolidation, focusing on the

role of QR2 in the anterior insular cortex during a novel taste learning task. Previous work from their lab

identified QR2 as an important removable constraint for novel taste memory formation, and this paper

refines the cellular identity responsible for the effect. The authors conclude 1) insula QR2 is reduced in

both excitatory and inhibitory cells following novel taste exposure, 2) the reduction in QR2 is most

prevalent in SST+ interneurons (INs), 3) reducing QR2 decreases SST+ IN excitability, and 4) artificially

reducing QR2 in SST+ INs enhances taste memory. The experiments are well designed and the results

are novel. However, there are some concerns that reduce my enthusiasm, as detailed below:

Major concerns:

- It appears that analysis of relative QR2 in Figures 1 & 2 use each individual cell’s relative expression

intensity as the n-values rather than determining a quantitative metric for each animal, such as total

# of cells counted or average relative intensity of QR2 expression per animal (as done in Figure 4). The 3

current metric falsely inflates the n-values, resulting in highly significant results and small standard

error of the mean. Additionally, the differences in the number of cells which express QR2 mRNA

transcript appear to differ greatly between groups - this should be quantified and interpreted.

The reviewer’s suggestion that rather than using pooled cell populations, each animal should be

evaluated by the mean measurement of its cells in order to avoid inflated statistical interpretation is an

excellent suggestion, and has been applied throughout the manuscript results and methods sections,

excluding one part (Fig. 3A-C) which explicitly aims to compare cells, and not animals. This revision

further sharpens the findings - namely, inhibitory SST neurons show a significant reduction in QR2,

which is much less prominent in excitatory cells - and further focuses the QR2 pathway onto inhibitory

cells, and SST neurons. We thank the reviewer for this excellent suggestion.

With regards to the variability in cell count, the method used utilizes fresh-frozen brains. Unfortunately,

the procedure of transferring un-fixed, 20 micron thick, fresh frozen brain slices onto slightly-below room-temperature glass slides causes damage to the slices in some instances, and so slices were lost

during this process. This resulted in some groups having slightly more/less animals, or slices per animal.

The sampling, however, was random, and the experiment was blind. The cells measured within the

surviving slices were not defined by QR2 signal, but cell marker (Gad1, Slc17a7, Sst, Pvalb, Vip), ensuring

a robust cell definition and count, within which the QR2 expression level was then measured.

Additionally, whether there were fewer or more animals/cells did not affect the outcome, as can be

seen in Fig. 1. Familiar water has the highest cell count and familiar saccharin the lowest, but both

contained cells expressing more QR2 than the novel saccharin group.

- For the electrophysiology, it is mentioned in the text that other intrinsic properties measured did not

differ between the groups, but this is not presented anywhere in the paper (it is mentioned that they

are in Figure 3-1 and 3-2, but these are not present). Please add which properties were measured and

include the figures, including the mentioned change in resting membrane potential.

Reviewer 1 raises an unfortunate formatting error, which prevented access to important data that was

therefore not available to them. Since these data have been highlighted by the reviewer as necessary,

we have introduced them as full figures to the manuscript (figures 5 and 6), including statistical analysis

and descriptive results within the new figure legends, with reference to these new figures within the

results section.

- For the novel taste memory task, some of the control groups (Figure 4D & 4F) present preference at

chance levels, thus indicating that this task is not necessarily producing much of a memory at all, or at

least a relatively weak one. While the manipulations to decrease QR2 expression present compelling

evidence that a reduction in this pathway in SST INs can augment learning, it would be nice to show the

reverse of this outcome - that blocking QR2 from reducing causes memory impairment.

Reviewer 1 makes an excellent observation, namely that in control groups there is no preference for the

novel taste, and thus likely demonstrates very weak, if any learning. While this observation is true for

certain paradigms such as NOR, where a familiar item elicits less exploration from the animal compared

to a novel object - with taste this is different and not the case. In taste learning, novelty of a taste elicits

caution, as an innate, evolutionarily conserved trait. The term for this very careful sampling of new

foodstuffs with unfamiliar tastes is “neophobia", and since this is such a well conserved and described 4

trait, it has served as a central pillar in taste memory research for many decades, in model animals and

humans (Green and Parker, 1975; Birch et al., 1987; Yiannakas and Rosenblum, 2017). As an example,

presented below (Figure 1) is data from an experiment using WT mice, in which the process of

familiarisation, and of learned safety of the palatable taste (since they consume the liquid with no

adverse effect), elicits an attenuation of neophobia. Therefore, results for the control groups in the

present paper are on par with the literature and expected results (Figure 1, trial 1) and represent the

expected outcome using these animals. However, the reviewer is still right in pointing out this is weak

learning. Attenuation of neophobia is a form of incidental learning, and has no associative stimulus or

consequence. It therefore normally requires multiple exposures to become well remembered. Only by

having exceptionally good memory of the very first encounter of the taste is attenuation accelerated

during trial 1 (which is the time point we used in the present manuscript, being the time point in which

changes to memory strength are most noticeable and measurable), since the mouse requires fewer

exposures to form a stable memory and familiarisation of the taste. Therefore, the results described in

this manuscript represent classic incidental novel taste learning results and behavioural outcomes.

With regards to preventing QR2 reduced expression and impairment of taste memory, there are already

a number of papers that show this (Brouillette and Quirion, 2008; Benoit et al., 2010; Hashimoto and

Nakai, 2011; Rappaport et al., 2015; Gould et al., 2020). Additionally, in our previous publication (Gould

et al., 2020) we detail how ACh controls QR2 expression in the aIC using, amongst others, scopolamine

(a very well-known method for preventing memory for a taste (Gutiérrez et al., 2003) and causing

elevated QR2 expression) and demonstrated that inhibiting QR2 rescues scopolamine induced taste

amnesia, pointing to reduced QR2 levels in aIC as critical for intact novel taste memory formation, and

that elevated QR2 levels impede this process.

Figure 1. Attenuation of Neophobia in Wild-Type Mice. 5

Mice were trained to drink water from pipettes during a period of 20min/day for 3 days, and on day 4

received saccharin (novel taste) for the first time. Following a further day in which water was given, on

days 6-8 the mice received both water and saccharin (test days 1-3). Preference for saccharin was

calculated as a percentage of saccharin consumed from the total volume the mice drank, in each day. A

significant increase in preference was seen over successive exposures (test of within subject effects,

df=2, F = 13.98, p= 0.001; Bonferroni multiple comparisons test: Test Day 1 vs. Test Day 2, p= 0.018; Test

Day 1 vs. Test Day 3, p= 0.015; Test Day 2 vs. Test Day 3, p= 0.526).

Benoit CE, Bastianetto S, Brouillette J, Tse YC, Boutin JA, Delagrange P, Wong TP, Sarret P, Quirion R

(2010) Loss of quinone reductase 2 function selectively facilitates learning behaviors. J Neurosci

30:12690-12700.

Birch LL, McPhee L, Shoba BC, Pirok E, Steinberg L (1987) What kind of exposure reduces children’s food

neophobia?. Looking vs. tasting. Appetite 9:171-178.

Brouillette J, Quirion R (2008) Transthyretin: A key gene involved in the maintenance of memory

capacities during aging. Neurobiol Aging 29:1721-1732.

Gould NL, Elkobi A, Edry E, Daume J, Rosenblum K (2020) Muscarinic-Dependent miR-182 and QR2

Expression Regulation in the Anterior Insula Enables Novel Taste Learning. eneuro:ENEURO.0067-

20.2020 Available at: http://eneuro.org/lookup/doi/10.1523/ENEURO.0067-20.2020 [Accessed

March 31, 2020].

Green KF, Parker LA (1975) Gustatory memory: Incubation and interference. Behav Biol 13:359-367

Available at: https://linkinghub.elsevier.com/retrieve/pii/S0091677375914169.

Gutiérrez R, Téllez LA, Bermúdez-Rattoni F (2003) Blockade of cortical muscarinic but not NMDA

receptors prevents a novel taste from becoming familiar. Eur J Neurosci 17:1556-1562 Available

at: http://doi.wiley.com/10.1046/j.1460-9568.2003.02608.x [Accessed September 26, 2019].

Hashimoto T, Nakai M (2011) Increased hippocampal quinone reductase 2 in Alzheimer’s disease.

Neurosci Lett 502:10-12 Available at: http://dx.doi.org/10.1016/j.neulet.2011.07.008.

Rappaport AN, Jacob E, Sharma V, Inberg S, Elkobi A, Ounallah-Saad H, Pasmanik-Chor M, Edry E,

Rosenblum K (2015) Expression of quinone reductase-2 in the cortex is a muscarinic acetylcholine

receptor-dependent memory consolidation constraint. J Neurosci 35:15568-15581.

Yiannakas A, Rosenblum K (2017) The insula and taste learning. Front Mol Neurosci 10.

Reviewer #2 (Rationale for Significance Rating for Authors (Required)):

please see my main review

Reviewer 2 acknowledges that the QR2 pathway is a part of intracellular signalling involved in memory

consolidation, and that the current manuscript contributes to our understanding of this process. The

reviewer also notes that all supplementary data is missing, to the detriment of the manuscript, and

further speculates on electrophysiological characterisations that would have benefitted the paper, 6

which were omitted by the lack of supplementary data availability. Reviewer 2 also notes that no follow-up experimental studies were done to establish which ion channel is being affected by QR2. Finally, the

reviewer questions the efficacy of the shRNA viruses targeting QR2. Much of what reviewer 2 notes has

brought to light the importance of the supplementary information, requiring their inclusion as full

figures, helping form a much more robust manuscript. Additionally, their remarks have helped improve

the writing, particularly concerning the use and analysis of RNAscope FISH, while other comments point

to the nascent nature of this emerging subject and planned future studies.

Reviewer #2 :

A few years ago, Quinone reductase 2 (QR2) has been shown by this group to be reduced in expression

in the learning paradigm of conditioned taste aversion, downstream to activation of muscarinic ACh

receptors in the anterior insular cortex (aIC) (Rappaport et al. 2015). More recently, this lab also showed

that QR2 is coupled to changes in excitability in hippocampal interneurons (Gould et al. 2020b), and

these authors have further elaborated the role of QR2 in the aIC, possibly involving changes in the redox

state of Kv2.1 (Gould et al. 2020a). Thus, the current knowledge suggests that QR2 is part of an

intracellular signaling pathway involved in memory consolidation. In the current paper, Gould et al.

follow-up on the role of QR2 in “novel taste learning” in the aIC. They compare mRNA levels of QR2 with

ISH (RNAscope?) and find that QR2 mRNA is reduced in principal (excitatory) neurons and in

somatostatin (SST)-INs (Figs 1, 2; but see points 1, 2). Using patch-clamp recordings of SST+ cells in SST Cre mice, and of excitatory neurons, they find that the “excitability” of SST-INs is “reduced” (Fig. 3).

However, a more detailed analysis would have been worthwhile - the initial slopes in the relations

between AP-frequency and injected current seems unchanged, and the “reduced excitability” mainly

seems to show up with high current injections; was this phenomenon some sort of “depolarization

block”? Furthermore, unfortunately, no attempt was made to study which ion channels are involved in

the changed AP-firing. In a final Fig. 4, the expression of QR2 is down-regulated by shRNA approaches in

various neuron types of the aIC, and it is concluded that QR2 signaling in SST- and excitatory neurons,

but not in PV-INs, is involved in learning a novel taste. However, it was unclear why shRNA was quite

inefficient in reducing the expression of QR2 mRNA levels (by only about 20 - 30% at most; much less in

PV- and SST-INs; Fig. 4). Taken together, given the recent findings by this group, the current paper

contributes incrementally to what is known about the role of QR2 in learning.

Reviewer 2 rightly asks for further details regarding the electrophysiological recordings in the cells,

which were removed by the portal submission process. These have now been added within the

manuscript as figures 5 and 6.

Regarding assessment of which ion channel is affected by QR2 inhibition - since QR2 is a redox

regulator, the effect of QR2 inhibition may cover a number of channels, and these may alter in different

cell types. Therefore, in order to appropriately study which channels are effected in each cell would

constitute experimentation beyond the scope of the current paper, which aimed to evaluate cell-type

and location of QR2 removal, as well as intrinsic property evaluation to determine a cellular phenotype

within said cells. Future work will reveal the exact ion channel/s and intracellular machinery necessary

to generate this effect in greater detail, in the different, relevant cell types.7

The measurement of QR2 mRNA by qPCR requires precise excision of a small region of the mouse IC,

without any visual guidance to the injection site. Therefore, the tissue removed invariably contains

uninfected brain and blood tissue, which inevitably reduce the delta observed. Therefore, when using a

general promoter within a viral vector to knock-down QR2 expression, results beyond a 30% reduction

in the brain are rare, yet still effective (Rappaport et al., 2015; Gould et al., 2020a, 2020b). When using

cell-specific Cre expression of the vector, the PV, VIP or SST cells that are included within the punched

tissue constitute a tiny fraction (very roughly 2-6%) of the total neuronal population. Therefore, the

expected change seen from this population cannot itself exceed far beyond the observed reduction.

With vGlut or GAD, on the other hand, the proportion of cells is far higher, so a clearer reduction can be

measured. However, by measuring mCherry reporter and the small reduction in QR2 expression, one is

able to validate, along with slice imaging, as well as one can, the efficacy of the viral manipulation (since

RNAscope of these brains is not viable due to the fluorescent reporter genes expressed in situ, that are

orders of magnitude stronger than the probe signal, and include similar colours).

Specific points.

1) The writing of the paper should be improved, explaining the type of analyses more accurately in all

parts of the ms. For example, how exactly were the bar graphs of “relative QR2 mRNA” constructed?

(e.g. Fig. 1D-F). Was this the intensity of a fluorescence value (indicating QR2 mRNA levels) in individual

cells, and the averaged? How was the normalization done? The description in Methods, Results and

Figure legends should be improved.

Reviewer 2 suggests that the methods for QR2 expression level measurement and analysis shown in the

figures should be better written. The relevant sections in the paper have been revised, including the

results section, figure legends and the methods section (lines 154-188), improving the clarity of the

manuscript for which we thank the reviewer for their comment. The reviewer asks for a clarification

regarding how bar graphs of relative QR2 mRNA were constructed. This we have also added to the

manuscript, in the aforementioned sections. Briefly, RNAscope FISH labelling of cell type and QR2 was

done. Mean QR2 expression within every cell type was measured per mouse, and this value was then

divided by the average of the means from the familiar (water) control group. This results were then

compared across groups.

2) In Fig. 2, example images of QR2 ISH in the three types of interneurons should be shown - we only get

to see the bar graphs for “PV", VIP", “SST”. Also, in this series of experiments, how many fluorescence

channels were you able to analyze at once? i.e. were the QR2 levels in all 3 cell inhibitory interneuron

types measured in the same sections / same mice?

In Fig. 2 (now Fig. 3), the RNAscope FISH images are given in panel D of the figure, in greyscale. This was

done in order to clearly show the location and number of each cell type within the aIC, as detected with

this method. We have clarified this by revising the text in the results section and the figure legend.

In any given RNAscope assay, up to 3 probes can be used at once

(https://acdbio.com/rnascope%C2%AE-fluorescent-multiplex-assay), and are available only in certain

combinations (depending on C1, C2 and C3 probe design, which also depends on the probe target itself,

e.g. - weakly/strongly expressed targets require different probe allocation, since some probes allow

better labelling for weaker targets and vice-versa). Therefore, multiple slices from the same mice in all

the groups were used, in order to collect the data across all five cell types. In order for the sections to 8

represent the aIC specifically and uniformly, sequential 20um slices were used within the same area of

the animal’s aIC (bregma AP 1.25 - 0.25). QR2 was measured in GAD and vGlut expressing neurons on

the same slice; in VIP and PV on the same slice; and in SST alone.

3) For the analysis of “mAHP” in Fig. 3, suitable example traces, and how the measurements were done,

should be shown.

4) The authors refer to Extended data Figures (“Fig. 1-1"; l. 256; “Fig- 3-1” and “Fig. 3-2"; l. 297), which,

unfortunately, were not visible for this reviewer on the J. Neuroscience ms site.

Regarding points 3-4, the unfortunate omission of all the supplementary data has led to these open

questions and comments. We have therefore incorporated Fig.’s 1-1, 3-1 and 3-2 as full figures 2, 5 and

6, respectively, with relevant reference in the results section and figure legends.
